# Are We Adding Pain-Free Years to Life? A Test of Compression Versus Expansion of Morbidity

**DOI:** 10.1093/gerona/glae157

**Published:** 2024-06-15

**Authors:** Zachary Zimmer, Feinuo Sun, Amber Duynisveld

**Affiliations:** Department of Family Studies & Gerontology and The Global Aging Research Initiative, Mount Saint Vincent University, Halifax, Nova Scotia, Canada; Department of Kinesiology, University of Texas at Arlington, Arlington, Texas, USA; Department of Family Studies & Gerontology and The Global Aging Research Initiative, Mount Saint Vincent University, Halifax, Nova Scotia, Canada; (Medical Sciences Section)

**Keywords:** Chronic pain, Health expectancy, Longevity, Longitudinal analysis, Multistate life tables

## Abstract

**Background:**

There has been debate regarding whether increases in longevity result in longer and healthier lives or more disease and suffering. To address the issue, this study uses health expectancy methods and tests an expansion versus compression of morbidity with respect to pain.

**Methods:**

Data are from 1993 to 2018 Health and Retirement Study. Pain is categorized as no pain, nonlimiting, and limiting pain. Multistate life tables examine 77 996 wave-to-wave transitions across pain states or death using the Stochastic Population Analysis for Complex Events program. Results are presented as expected absolute and relative years of life for 70-, 80-, and 90-year-old men and women. Confidence intervals assess significance of differences over time. Population- and status-based results are presented.

**Results:**

For those 70 and 80 years old, relative and absolute life with nonlimiting and limiting pain increased substantially for men and women, and despite variability on a wave-to-wave basis, results generally confirm an expanding pain morbidity trend. Results do not vary by baseline status, indicating those already in pain are just as likely to experience expansion of morbidity as those pain-free at baseline. Results are different for 90-year-olds who have not experienced expanding pain morbidity and do not show an increase in life expectancy.

**Conclusions:**

Findings are consistent with extant literature indicating increasing pain prevalence among older Americans and portend a need for attention to pain-coping resources, therapies, and prevention strategies.

Longevity has been increasing in the United States for decades. According to life table data from the Centers for Disease Control and Prevention (CDC) (1) the probability of living from birth to 70 was 57% in 1950, reached 71% by 1990, and 78% by 2018. At the same time, the probability that those reaching 70-years-old survived to age 85 was about 28% in 1950, got to 45% by 1990, and 55% by 2020. These figures imply that increasingly more Americans live to an old age, and more so, those who live to an old age are increasingly likely to survive to extreme old age. Consequently, the number of years of life expected to be lived in old age has substantially increased, and the very old are the fastest-growing segment of the U.S. population.

For some time now gerontologists have pondered the implications of these increasing survival probabilities, particularly with respect to its impact on population health, health resources, and policy (2,3). Much of this discourse is rather pessimistic, imagining increasing financial obligations and individual hardships accompanying populations living to very old ages. The cynicism has resulted in some dramatic public statements. A well-publicized article in The Atlantic by eminent physician and health policy expert Ezikiel Emmanuel (4) titled “Why I Hope to Die at 75,” suggested that declining productivity, increasing disease, disability, and chronic pain that are typical attributes of the very old means families, societies, and individuals are better off “if nature takes its course swiftly and promptly.” In a recent podcast interview, alternative medical advocate and health philosopher Deepak Chopra said about modern medicine’s ability to increase the length of lives: “It doesn’t promote life; it promotes misery” (5).

These arguments echo a debate about the implications of longevity improvements prompted by theoretical frameworks that posit opposing population health outcomes. Often attributed to Ernest Gruenberg (6), the expansion of morbidity hypothesis postulates that while modern medicine is capable of removing the sequelae of disease, it results in individuals surviving to ever-increasing old ages suffering from increasing morbidity and frailty. In contrast, the compression of morbidity hypothesis ascribed to James Fries (7) is much more optimistic, proposing that when life expectancy is extended to natural limits, morbidity becomes compressed into a short period at the end of life, reducing the proportion of life lived in unhealthy states. Although Fries’ argument originally postulated that human lifespan was fixed, current understanding of the argument suggests that as longevity expands, unhealthy years of life contract (8,9). The bottom line rests upon the degree to which gains in years of life translate into frail, disabled, and painful years; expansion argues for increasing years and shares with morbidity, while compression argues the opposite. There are also compromised positions. Increases in life expectancy in old age may be accompanied by proportionate changes in share of life with morbidity, or expansion in milder but not more extreme states of morbidity, situations referred to as dynamic equilibrium (10).

A fair number of studies have tested whether the U.S. population has been experiencing a compression or expansion of morbidity or some compromised situation (11,12). Health expectancy frameworks, whereby a life table approach is used to separate life expectancy into years expected in different states of health, are commonly used for these studies (9,13). If life expectancy increases more than life expected in a healthy state, in both relative and absolute terms, then an expansion of morbidity is taking place. In contrast, if life expectancy increases less than life in a healthy state, it indicates a compression.

Studies that test for compression using these methods often conceptualize morbidity as disability or functional limitation. Many, though certainly not all, of these studies have tended to fall on the compression side of the debate for the United States, especially with respect to severe disability (14–17). Studies examining life expected with chronic disease have been far less encouraging, often finding that increases in life translate into more years with disease morbidity (18–20). As such, there may be differences between increases in chronic disease morbidity versus increases in disability morbidity. In a comprehensive study, Payne (21) examined successive cohorts and alternate measures and generally found an expansion with respect to chronic disease and an equilibrium with respect to disability. Crimmins et al. (22) in contrast found a compression with respect to cognitive impairment in the United States. The net of these and other similar studies suggest old-age life expectancy has generally been increasing in the United States, but whether years of life gained are lived with more or less morbidity is decidedly mixed. Findings differ depending upon the measure used to define morbidity, the population being studied, and whether compression is defined in absolute or relative terms.

The current study examines whether there was a compression or expansion of pain morbidity over a 25-year period, 1993–2018, among old-age adults in the United States. To highlight trends for the older adults, the study focuses on an upper age range, ages 70 and up, and categorizes less severe and more severe states of pain. Few studies have examined life expectancy with and without pain (23,24), and none have examined trends in pain expectancy. Yet, there are good reasons for considering pain as a critical measure of population health and a useful indicator for expansion or compression of morbidity (25). Pain has been recognized as a global public health concern given its implications for productivity and life quality (26,27). The Global Burden of Disease has highlighted pain as being among the world’s most prevalent health conditions (28). Furthermore, pain is closely linked to disability and functional limitation, measures more often referred to in health expectancy studies (29–32).

## Method

### Data

Data come from Health and Retirement Study (HRS) (33,34). But, in order to monitor trends in pain expectancy over the longest possible period for a population aged 70+, the study begins with the 1993 Asset and Health Dynamics among the Oldest Old (AHEAD), which merged with and became part of HRS in 1998. HRS and AHEAD are public-use data sets produced and distributed by the University of Michigan with funding from the National Institute on Aging (grant number NIA U01AG009740). AHEAD involved extensive surveys of the American population aged 70+ and included several questionnaire items assessing pain status. It was administered in 1993 and 1995. HRS involves the population aged about 50+ and includes the same questions for assessing pain as were in the 1993 AHEAD. From 1998 onward HRS has been followed up every 2 years. HRS adds individuals to their sample every 3 waves, partly to substitute for losses to follow-up and deaths, thus regenerating the sample size.

The health expectancy method employed in the study uses a multistate life table technique (MSLT), which requires transition probabilities as input. The analysis uses 2-year wave-to-wave transition probabilities calculated from a baseline and follow-up observation. Each wave with a recorded pain state is a baseline and the subsequent wave, with a recorded pain state, or a death, is a follow-up. Every wave from 1993 to 2016 is considered a baseline except for 1995, which is omitted because of consistency, for instance, it has a 3-year instead of a 2-year transition period and a sample aged 72+ instead of 70+. Every wave until 2018 is a follow-up. The study does not go further than 2018 because the 2020 data, though available, is influenced by the coronavirus disease-2019 pandemic. Therefore, the study monitors trends over 11 wave-to-wave transition periods, each considering a community sample aged 70+. Across the 11 periods, there are 77 996 valid transitions recorded. Across waves, there are 188 missing pain responses at baseline and an additional 4 917 observations with missing follow-up information. These observations are omitted from the expectancy calculations. Details on *N*’s by sex and baseline waves are shown in Supplementary Table 1. Results shown in this study apply HRS individual sample weights at each baseline to adjust the study population to be generalizable to noninstitutionalized Americans aged 70+ nationwide. Observations missing at follow-up are removed prior to any analysis and weights are adjusted based on the remaining valid sample.

### Measures

Health and Retirement Study and AHEAD surveys include the following questions about pain: “are you often troubled with pain?” and for those who say yes, “does the pain make it difficult for you to do your usual activities such as household chores or work?” The 2 questions are used to categorize individuals at any of the 11 baselines into 3 pain states: no pain, nonlimiting pain, and limiting pain. (In most waves, there is a third question about intensity of the pain, although this question does not appear in the 1995 version.) The categorization of limiting pain is particularly important since it indicates a more severe degree of pain and one that correlates well with functional limitation and/or disability. Pooling data across all baselines, about 67% are categorized as no pain, 12% as nonlimiting pain, and 21% as limiting pain. Across all 11 follow-ups, individuals are categorized into 1 of these 3 pain states or as deceased. Dates of death are monitored by HRS and provided in its file tracker (35). Death rates in HRS have been found to be valid and reliable (36). Pooling data across all follow-ups, at follow-up, 59% are pain-free, 11% have nonlimiting pain, 19% have limiting pain, and 11% died. In addition to pain, the study considers age and sex as recorded at baseline. 58% of the recorded transitions are from women. The average age across all baselines is 78.0 with a standard deviation of 6.1. 64% are aged 70–79 at baseline, 31% are 80 to 89, and 5% are 90 and older.

### Statistical Analysis

Estimation uses the Stochastic Population Analysis for Complex Events (SPACE) program (37,38) to calculate life expectancies in 3 pain states: pain-free life expectancy (PFLE), nonlimiting-pain life expectancy (NLPLE), and limiting-pain life expectancy (LPLE). Simple subtraction provides additional estimates of limiting pain-free life (LPFLE) expectancies. Estimates are presented in 2 forms. Absolute estimates are net years in any particular state, with the sum across states being total life expectancy (TLE). Relative estimates are the proportion of total life in any particular state, with the sum across states totaling one. In addition, expectancies are estimated for several groupings. First, “population-based” estimates consider the total population regardless of baseline state. “Status-based” estimates separate the population by baseline state. Status-based estimates permit a look at whether changes in pain expectancy for the total population are consistent for those who begin an observation period pain-free, with nonlimiting pain, or with limiting pain.

Stochastic Population Analysis for Complex Events produces these estimates using a MSLT (13). An advantage of SPACE over other MSLT techniques is its ability to derive standard errors and confidence intervals for relative and absolute estimates. The MSLT approach constitutes 2 stages. In the first, information about each wave-to-wave transition is used in a multinomial regression equation to fit the probability of transitions from any single baseline state to 1 of 4 follow-up states. For this analysis, this regression predicts outcome pain status based on baseline status, age, and sex, using HRS weights to account for survey design. That is, for any baseline, each observation is classified as pain-free, nonlimiting pain, or limiting pain. At subsequent follow-up waves, the observation is classified as being in 1 of these 3 states or deceased. This means that across subsequent survey waves, an individual who does not have pain can remain pain-free, deteriorate to one of pain states, or die. An individual in a state of pain, either nonlimiting or limiting, can remain in pain, die, or improve to being pain-free. The descriptive results will show that both wave-to-wave deterioration and improvement are possible outcomes. In the second stage, a microsimulation is conducted, beginning with a hypothetical cohort distributed into initial pain states. Transition probabilities are used to estimate individual outcomes on a year-to-year basis until the hypothetical cohort dies out. Summary statistics estimate average length of life of a cohort in each state. Bootstrapping derives standard errors. This study uses a relatively large bootstrap of 300. Confidence intervals consider the 2.5th to the 97.5th percentile for the bootstrapped distributions.

Estimates of absolute years, which are net years of life expected, and relative years, which are percent of remaining life expected, in each pain state, are calculated for each of the 11 baseline waves and displayed for ages 70, 80, and 90. Trends in pain expectancy, and whether they suggest expansion or compression of pain morbidity, are assessed in several ways. First, estimates are examined to assess if they are falling or rising over time. Second, an average year-to-year change for each pain state is determined using a linear formula. For absolute years, this is a simple ordinary least squares regression (OLS). Since relative years are expressed as a proportion, average change is assessed using a fractional logit regression, which expresses the change in log odds. Third, standard errors determine 95% confidence intervals for each estimate at each wave and subsequently assess whether any estimate is significantly smaller or larger in comparison to the 2016 baseline estimate, which is the last estimate in the series of 11. If pre-2016 estimates are significantly larger, it indicates a declining trend. Evidence of a rising trend exists if pre-2016 estimates are significantly smaller than the 2016 estimate. The 3 assessments together are used as evidence for compression or expansion of morbidity. For instance, considering absolute LPLE, a compression is suggested by declining estimates over time, a negative linear trend, and significantly larger estimates in pre-2016 waves in comparison to 2016. The opposite suggests an expansion.

Health expectancy analyses tend to produce huge amounts of potentially meaningful output. Much of this is delegated to tables in Supplementary Materials as noted subsequently. Presented in this study are tables and figures that best summarize that output. Supplementary analyses were conducted to compare the 6.3% of the total sample that was dropped from the analysis due to loss-to-follow-up. These analyses indicated that the missing sample is no different from the valid sample with respect to variables that the HRS uses to compute sample weights.

## Results

### Wave-to-wave Pain Transitions

Table 1 shows wave-to-wave transition probabilities for men and women aged 70+ aggregated across all baselines. Follow-up pain state probabilities are highly dependent on baseline state as expected. For instance, men who are pain-free at baseline are most likely to be pain-free at follow-up (75.0%); those with limiting pain at baseline are most likely to have limiting pain at follow-up (45.9%). But there is also substantial movement across states. For men, for instance, 7.3% pain-free at baseline report nonlimiting pain at follow-up, and 6.8% report limiting pain. Forty percentage of men with nonlimiting pain recover and are pain-free at follow-up, and 22.8% with limiting pain are pain-free at follow-up. Women have slightly worse pain outcomes but are less likely to die. The probability of dying is related to baseline state, but the difference is between those with limiting pain and pain-free or nonlimiting pain. For men, 10.9% of those pain-free and 11.5% of those with nonlimiting pain die before follow-up compared to 18.7% of those with limiting pain. For women, 8.4% of those pain-free and 8.1% of those with nonlimiting pain die compared to 12.7% of those with limiting pain.

**Table 1.** Distribution of Wave-to-wave Pain Status Transitions in the HRS, 1993–2018

	Baseline status							
	No pain (*N* = 52 094)		Nonlimiting pain (*N* = 9 649)		Limiting pain (*N* = 16 266)		Total (*N* = 77 996)	
Outcome status	*N*	%	*N*	%	N	%	N	%
Men								
No pain	17 406	75.0	1 614	40.1	1 207	22.8	20 227	62.2
Nonlimiting pain	1 697	7.3	1 197	29.7	666	12.6	3 560	10.9
Limiting pain	1 572	6.8	752	18.7	2 425	45.9	4 749	14.6
Died	2 541	10.9	465	11.5	990	18.7	3 996	12.3
Total	23 218	100.0	4 028	100.0	5 288	100.0	32 521	100.0
Women								
No pain	21 290	73.7	2 231	39.7	2 250	20.5	25 771	56.7
Nonlimiting pain	2 331	8.1	1 559	27.7	1 188	10.8	5 078	11.2
Limiting pain	2 837	9.8	1 374	24.4	6 144	56.0	10 355	22.8
Died	2 418	8.4	457	8.1	1 396	12.7	4 271	9.4
Total	28 876	100.0	5 621	100.0	10 978	100.0	45 475	100.0

*Note*: HRS = Health and Retirement Study.

### Population-Based Expectancy Estimates

Absolute expectancy estimates for the total population by baseline wave are shown in Table 2. Confidence intervals are shown in Supplementary Table 2. Estimates for any wave between 1993 and 2014 are bolded and italicized if significantly higher than the 2016-year estimate, indicating a declining trend, and bolded only if significantly lower than the 2016-year estimate, indicating a rising trend. For instance, men aged 70 in 1993 are estimated to live 11.47 years pain-free. This is significantly higher than the 2016 estimate of 9.60, indicating an absolute decline in PFLE. For 70-year-old men, the estimates in 2002, 2006, and 2010 are also significantly higher than in 2016, while no estimates are significantly lower. The column labeled β describes the average year-to-year change based on an OLS trendline. For PFLE for men aged 70, it is −0.053, indicating that on average PFLE declines by 0.053 years from year to year. Over the full observation period, there is an expected absolute decline in PFLE of 23 times this coefficient, or 0.22 years. Although this is substantial, the trendline is not statistically significant at *p* < .05.

**Table 2.** Estimated Expectancies in Various Pain States at Aged 70, 80, and 90, by sex, 1993–2016, and trend based on OLS

Sex	Age	Estimate	1993	1998	2000	2002	2004	2006	2008	2010	2012	2014	2016	β
Men	70	PFLE	***11.47***	9.21	9.71	***10.94***	9.82	***11.69***	9.31	***11.40***	9.41	8.78	9.60	−0.053
		NLPLE	**1.24**	**0.94**	**1.32**	**1.65**	**1.58**	**1.69**	**2.12**	**1.96**	2.43	**2.31**	2.76	0.072^**^
		LPLE	**1.42**	**1.96**	**1.95**	**2.27**	**2.92**	**2.38**	**2.69**	**2.92**	**2.98**	**2.77**	3.51	0.077^**^
		TLE	**14.13**	**12.11**	**12.98**	**14.86**	**14.32**	15.75	**14.12**	16.28	**14.81**	**13.86**	15.87	0.096‡
	80	PFLE	6.08	5.86	5.58	***6.38***	5.75	***7.22***	**5.19**	***6.86***	5.86	5.24	5.72	−0.013
		NLPLE	**0.88**	**0.79**	**0.87**	**0.96**	**1.07**	**0.89**	**1.14**	**1.18**	**1.17**	1.32	1.45	0.026^**^
		LPLE	**1.09**	**1.33**	**1.43**	1.60	1.95	**1.30**	1.70	1.86	1.66	**1.46**	1.79	0.023*
		TLE	**8.05**	**7.98**	**7.87**	8.95	8.76	9.41	**8.03**	***9.90***	8.69	**8.02**	8.96	0.036
	90	PFLE	2.90	***3.60***	3.12	3.40	3.13	***3.90***	2.70	***3.69***	3.24	2.84	2.99	−0.006
		NLPLE	0.56	0.61	0.52	0.53	0.65	0.43	0.52	0.65	0.52	0.66	0.65	0.003
		LPLE	0.76	0.86	0.91	1.07	***1.24***	0.70	0.94	***1.09***	0.83	0.71	0.76	−0.004
		TLE	4.22	5.07	4.54	5.00	***5.02***	***5.03***	4.16	***5.43***	4.59	4.21	4.40	−0.007
Women	70	PFLE	***12.13***	10.81	***11.10***	10.94	10.31	***11.12***	**9.01**	***12.65***	**9.49**	**8.67**	10.36	−0.088‡
		NLPLE	**1.39**	**2.17**	**1.88**	**1.84**	**2.10**	**2.10**	**2.19**	**2.48**	2.89	2.52	2.83	0.056^**^
		LPLE	**3.12**	**3.74**	**3.85**	**4.31**	4.82	**4.45**	4.90	**4.51**	***6.18***	5.46	5.23	0.107^**^
		TLE	**16.64**	**16.73**	**16.82**	**17.10**	**17.23**	17.66	**16.10**	***19.64***	18.57	**16.65**	18.42	0.075
	80	PFLE	7.21	6.53	6.68	7.24	6.29	6.77	**5.69**	***7.98***	6.58	**5.58**	6.70	−0.026
		NLPLE	**0.95**	**1.24**	1.25	**1.15**	**1.09**	**1.22**	1.35	1.51	1.56	1.38	1.53	0.023^**^
		LPLE	**2.41**	**2.53**	**2.36**	2.59	3.01	2.79	2.67	2.93	***3.69***	3.12	2.96	0.040^**^
		TLE	10.57	**10.31**	**10.29**	10.98	**10.38**	10.78	**9.70**	***12.43***	11.83	**10.09**	11.19	0.037
	90	PFLE	3.90	3.50	3.59	4.37	3.43	3.45	**3.16**	***4.45***	4.15	**3.06**	3.76	−0.005
		NLPLE	0.62	0.63	0.77	0.67	**0.50**	0.63	0.70	0.83	0.71	0.65	0.71	0.004
		LPLE	1.73	1.65	1.33	1.46	1.65	1.61	1.34	1.73	***2.01***	1.66	1.47	0.003
		TLE	6.26	5.78	5.69	6.49	5.58	5.69	**5.20**	***7.01***	***6.88***	**5.37**	5.93	0.002

*Notes*: LPLE = limiting-pain life expectancy; NLPLE = nonlimiting pain life expectancy; PFLE = pain-free life expectancy; LE = total life expected; OLS = ordinary least squares regression.

2016 estimate is significantly higher than estimates if only bolded at *p* < .05.

2016 estimate is significantly lower than estimates bolded and italicized at *p* < .05.

^**^ *p* < .01; *.01 < *p* < .05; ‡.05 < *p* < .10.

For men and women, there are several clear patterns. First, for those aged 70 and 80, TLE was higher in 2016 than in most earlier years. Although trendlines are not necessarily significant, the overall movement is upward and many wave estimates are significantly lower than the 2016 estimate. Also, the estimated change in life expectancy in these data is fairly similar to CDC estimates, but life expectancy estimates are slightly higher; likely a function of HRS being a community-based sample (1). Second, extra years of life tend to come from adding years of nonlimiting and limiting pain. Increases in LPLE for women aged 70 are particularly large, with a year-to-year average increase of 0.107, or a 2.46 rise over a 23-year period. Third, PFLE estimates have been relatively stable over time. The net result is gains in TLE are mostly a function of NLPLE and LPLE increases, and NLPLE and LPLE are increasing over time, consistent with the expansion of morbidity perspective.

But trends do not hold for those aged 90. First, there has been little change in life expectancy. This is not necessarily unexpected as others have reported a slow-down in life expectancy gains among older adults in the United States (11); CDC estimates for life expectancy at age 90 show only minimal change in the last couple of decades; and the HRS sample is rather small at the very old ages resulting in a degree of variability. More importantly, PFLE, NLPLE, and LPLE for the 90-year-old group are relatively constant over time.

Relative trends are shown in Table 3. These are more robust than absolute trends. For men aged 70 and 80, there is a precipitous decline in relative PFLE and very sharp increases in the share of NLPLE and LPLE. For instance, in 1993, 70-year-old men could expect 81.2% of remaining life to be pain-free. This dropped to 60.5% by 2016, with a consistent wave-to-wave declining pattern. In contrast, 70-year-old men could expect 10.1% of remaining life with limiting pain in 1993 and 22.1% by 2016, with a clear wave-to-wave rising pattern. Women aged 70 in 1993 were expected to live 18.7% of their remaining life with limiting pain. By 2016, the proportion had risen to 28.4%. With respect to relative estimates then, those 70 and 80 years old clearly conform to the expansion of morbidity pattern.

**Table 3.** Estimated Proportion of Life Expected in Various Pain States at aged 70, 80, and 90, by sex, 1993–2016, and Trend Based on Fractional Logit Regression

Sex	Age	Estimate	1993	1998	2000	2002	2004	2006	2008	2010	2012	2014	2016	β
Men	70	PFLE	***0.812***	***0.760***	***0.748***	***0.736***	***0.686***	***0.742***	***0.660***	***0.700***	0.635	0.634	0.605	−0.041^**^
		NLPLE	**0.088**	**0.077**	**0.102**	**0.111**	**0.111**	**0.107**	**0.150**	**0.120**	0.164	0.166	0.174	0.041^**^
		LPLE	**0.101**	**0.162**	**0.150**	**0.153**	0.204	**0.151**	0.190	**0.179**	0.201	0.200	0.221	0.030^**^
	80	PFLE	***0.755***	***0.734***	***0.709***	***0.713***	0.656	***0.767***	0.647	0.693	0.674	0.653	0.639	−0.022^**^
		NLPLE	**0.110**	**0.099**	**0.110**	**0.107**	**0.122**	**0.095**	0.142	**0.119**	0.134	0.165	0.162	0.024^**^
		LPLE	**0.135**	0.167	0.181	0.179	0.222	**0.139**	0.212	0.188	0.192	0.182	0.200	0.013^**^
	90	PFLE	0.687	0.710	0.686	0.680	0.624	0.775	0.648	0.680	0.706	0.675	0.679	−0.002
		NLPLE	0.132	0.121	0.114	**0.105**	0.129	**0.086**	0.125	0.120	0.114	0.156	0.149	0.009
		LPLE	0.181	0.169	0.200	0.215	***0.247***	0.139	0.227	0.201	0.180	0.169	0.172	0.002
Women	70	PFLE	***0.729***	***0.646***	***0.660***	***0.640***	0.598	***0.630***	0.560	***0.644***	**0.511**	**0.521**	0.562	−0.033^**^
		NLPLE	**0.084**	**0.130**	**0.112**	**0.108**	**0.122**	**0.119**	0.136	**0.126**	0.156	0.151	0.154	0.025^**^
		LPLE	**0.187**	**0.224**	**0.229**	**0.252**	0.280	**0.252**	0.304	**0.230**	***0.333***	***0.328***	0.284	0.027^**^
	80	PFLE	***0.682***	0.633	0.649	***0.659***	0.605	0.628	0.586	0.642	0.556	**0.553**	0.599	−0.020^**^
		NLPLE	**0.090**	0.121	0.122	**0.105**	**0.105**	0.113	0.139	0.122	0.132	0.137	0.137	0.017^**^
		LPLE	0.228	0.246	0.229	0.236	0.290	0.259	0.275	0.236	***0.312***	***0.309***	0.264	0.015^**^
	90	PFLE	0.624	0.605	0.630	0.673	0.615	0.606	0.607	0.634	0.603	0.569	0.633	−0.005
		NLPLE	0.099	0.109	0.136	0.103	0.090	0.111	0.135	0.118	0.104	0.122	0.120	0.006
		LPLE	0.277	0.286	0.234	0.225	0.295	0.283	0.258	0.247	0.293	***0.309***	0.247	0.002

*Notes*: LPLE = limiting-pain life expectancy; NLPLE = nonlimiting pain life expectancy; PFLE = pain-free life expectancy.

2016 estimate is significantly higher than estimates if only bolded at *p* < .05.

2016 estimate is significantly lower than estimates bolded and italicized at *p* < .05.

^**^ *p* < .01; *.01 < *p* < .05; ‡.05 < *p* < .10.

Again, the patterns are not evident for those 90 years old. Women aged 90 in 1993 for instance expected to live 62.4% of remaining life pain-free and 27.7% with limiting pain. This compares to 63.3% and 24.7%, respectively in 2016, indicating very modest if any change, with few or no statistically significant differences between years.

The final column in Table 3 shows fractional logit regression coefficients describing the average trend in relative life across states. Interpreting the coefficient is facilitated by taking the exponent (EXP), which indicates odds. For instance, the odds of pain-free life for 70-year-old men declined by about (EXP −0.041) 4% on a year-to-year basis between 1993 and 2016. The odds of nonlimiting pain increased by a similar (EXP 0.041) 4% and the odds of limiting pain increased by (EXP 0.030) 3%. Looking at all coefficients, it is clear that there are significant changes in estimates of pain-free, nonlimiting, and limiting pain among 70- and 80-year-olds, while changes are nonsignificant for 90-year-olds.

**Figure 1.**
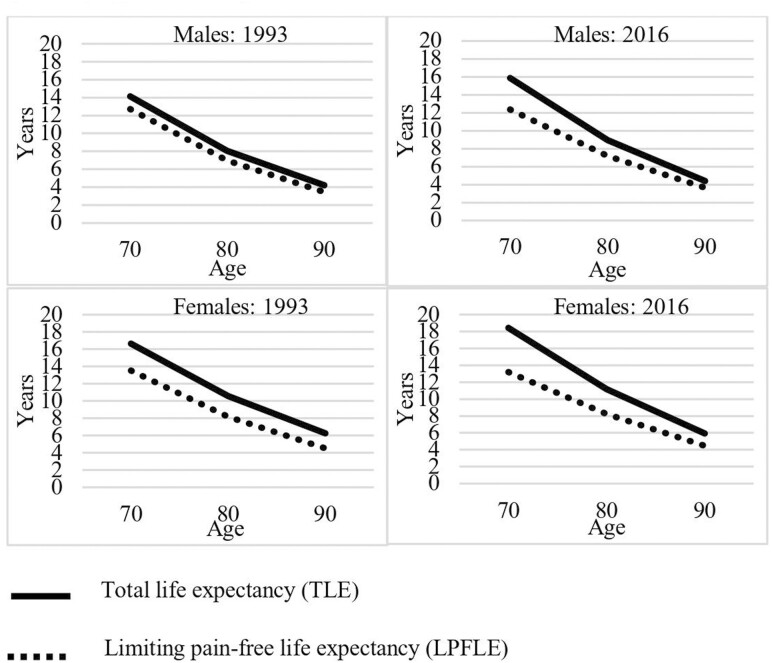
Total life expectancy (TLE) and limiting pain-free life expectancy (LPFLA), aged 70–90, by sex, 1993 and 2016.

These findings are shown in Figure 1 which plots and compares absolute estimates in TLE and LPFLE by age in years 1993 and 2016. Confidence intervals are shown in Supplementary Table 2. The space between solid and dotted lines, or the difference between TLE and LPFLE, is LPLE. At age 70, moving from 1993 to 2016, for both men and women, there is a widening of the gap between TLE and LPFLE, meaning more years to be lived with limiting pain. For example, for men, TLE at age 70 increased from 14.1 to 15.9 years, a gain of 1.8 years. LPFLE declined from 12.7 to 12.4 years. Therefore, LPLE increased from 1.4 to 3.5 years over the period, suggesting an expansion of pain morbidity. For women, TLE at age 70 increased from 16.6 to 18.4, or 1.8 years. LPFLE decreased from 13.5 to 13.2 years, meaning LPLE increased from 3.1 to 5.2 years; again, a clear indication of expansion.

By age 90, there is less change. TLE for men aged 90 increased by 0.2 between 1993 and 2016, from 4.2 to 4.4, while LPFLE increased by 0.2 years, from 3.5 to 3.7, meaning LPLE remained stable. For women aged 90, there was a slight decline in TLE from 6.3 to 5.9, while LPFLE remained stable at 4.5 years.

### Status-Based Expectancy Estimates

**Figure 2.**
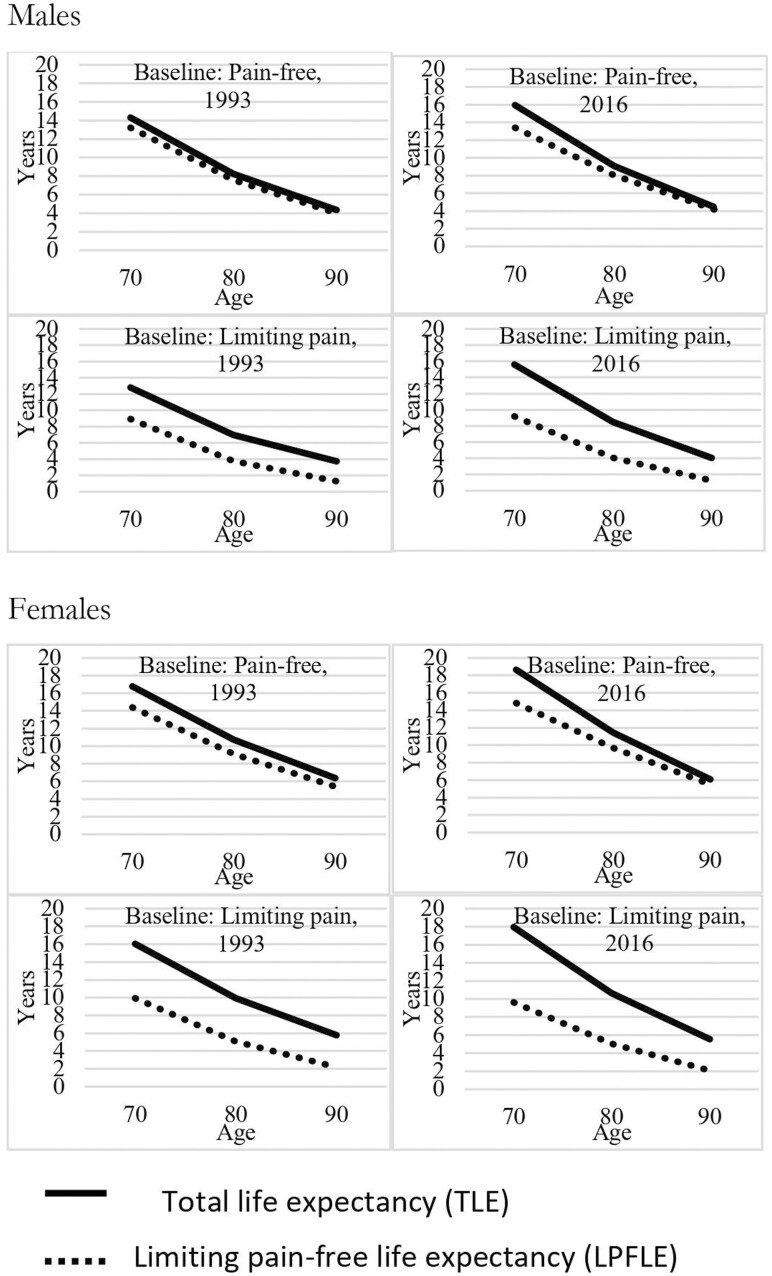
Total life expectancy (TLE) and limiting pain-free life expectancy (LPFLA), aged 70–90, by baseline pain status and sex, 1993 and 2016.

To assess whether conclusions are consistent across baseline states, Figure 2 shows the 1993 and 2016 estimates for TLE and absolute LPFLE for men and women, aged 70–90, using status-based estimates. Complete results are shown in Supplementary Table 3. Confidence intervals are shown in Supplementary Table 4. The findings here suggest the same phenomenon as for population-based estimates: expansion of morbidity at ages 70 and 80 but not at age 90. This can be seen in the increasing distance between TLE and LPFLE regardless of baseline state for 70- and 80-year-olds. For instance, men 70 years old and pain-free at baseline lived 14.3 total years of life in 1993 and 16.0 in 2013, an increase of 1.7 years of life. LPFLE increased from 13.2 to 13.4 years, or 0.2 years. Therefore, a large percentage of the total gain in life expectancy is a gain in LPLE. Similarly, for 70-year-old women, TLE increased from 16.8 to 18.7 years, or 1.9 years, while LPFLE increased from 14.4 to 14.8 years, or 0.4 years, meaning the remaining 1.5 years of life gained is LPLE.

Results for those with limiting pain at baseline are important for assessing the compression argument. If morbidity is compressed at the end of life, one would expect declines in LPLE for those with limiting pain at baseline. This however is not the case. For instance, 70 years old men with limiting pain at baseline lived 12.8 years in 1993 and 15.6 in 2016, for a gain of 2.8 years. LPFLE for this group increased from 8.9 to 9.2, meaning that the proportion of the 2.8-year life expectancy increase that is accounted for by life without limiting pain is only 0.3 years, and there was a 2.5-year gain in LPLE among those with limiting pain at baseline. For women aged 70 with limiting pain at baseline, TLE went from 16.0 to 18.0 for a net of 2.0 years. LPFLE declined from 9.9 to 9.6, meaning a 2.3-year increase in LPLE.

Again, the situation is different for the oldest adults. Gains in life were minor, and there was little change in years with limiting pain across baseline states. For men 90 years old, pain-free at baseline, TLE increased only from 4.4 to 4.5, and LPFLE went from 4.0 to 4.2 years. For 90-year-old women, TLE declined from 6.4 to 6.1 while LPFLE remained at 5.4. For those with limiting pain at baseline, men’s TLE rose from 3.7 to 4.1, a gain of 0.4 years, while LPFLE remained stable at 1.3, suggesting a small increase in LPLE of 0.4 years. Women’s TLE at age 90 when having to limiting pain declined from 5.8 to 5.6, while LPFLE declined from 2.2 to 2.1, so LPLE remained about stable.

## Discussion

Expanding longevity can prompt economic and social challenges (2,3). The degree to which increases in life expectancy results in increasing economic costs and human suffering depends largely on how extra years of life are lived. The compression of morbidity hypothesis presumes gains in life expectancy are gained in relatively healthy years. The expansion of morbidity hypothesis assumes life expectancy gains are at the cost of pain and suffering. Studies on whether the United States is experiencing compression, expansion, or some compromise, vary by indicator, period of study, and cohort (19). A recent comprehensive analysis by Payne (21), testing successive cohorts across several indicators, suggests expansion with respect to chronic conditions, compromise or equilibrium with respect to functionality, and some compression when considering self-rated health. In short, the evidence is highly mixed. The current study is the first to test compression versus expansion of pain morbidity for older Americans. Pain is a particularly consequential measure. It is also increasingly recognized as a critical indicator of population health (25,26,28,39) and is closely related to disability and the types of chronic diseases often considered by those testing for compression of morbidity (40).

The results of this study are unambiguous for 70- and 80-year-old men and women. They have experienced an expansion of pain morbidity. Individuals in the United States of this age are living longer lives, with extra years increasingly being years with both nonlimiting and more importantly, limiting pain. Even more critical for assessing compression or expansion, a larger proportion of remaining life for these individuals is increasingly being lived in pain states. The relative increase in life with pain is robust and meets several tests of statistical significance. The situation is different for those 90+. These individuals have not experienced compression or expansion.

Notwithstanding the more favorable findings for the oldest, the implications of the study are on balance alarming. They portend a need for increasing attention to pain-coping resources, therapies, and prevention strategies. It should be noted that several studies over the last decade or so have exposed rather distressing increases in pain prevalence in the U.S. population (41,42). The current study is consistent with these. Some of this has been attributed to rising rates of obesity and other modifiable risk factors (43,44), suggesting paths are available for reversing trends on the prevention side (45).

There are several other links that can be made between the current study and extant literature. Results here are inconsistent with trends in disability-free life expectancy, suggesting a possible disconnect between disability and pain (11). It may suggest that people are progressively able to cope with disability caused by pain. The results, however, are more in agreement with studies that have looked at chronic disease morbidity (21), which generally indicate people are living longer with diseases and perhaps consequently with the pain that accompanies them. It is also important to note that trends in health expectancy differ markedly in different parts of the world (9). Although the United States may be moving toward increasing years of pain morbidity, the same may not be the case across Europe, for example.

There are a number of limitations of this study. Pain expectancies may be highly dependent upon other characteristics, such as race and education, and thus trends may be variable demographically (24). It is beyond the scope of the current study to examine these, but future studies could expand the purview. On the technical side, the multistate life table methods used to calculate expectancies consider transition probabilities between 2 waves, with only 1 transition being observed between any 2 waves, based on the state reported at baseline and the state reported at follow-up. But there could be other unrecorded transitions between waves. For instance, an individual categorized as having limited pain at baseline and follow-up may have lived some of the inter-survey period pain-free. Inter-survey pain states are, however, not captured in the HRS. Although there are likely to be manifold changes in pain state between survey waves, it is also true that as long as the timing of these changes is random it should not affect expectancy estimates. Also, there is a fair bit of variation in the prevalence of pain in the study population from wave to wave. The interpretation of total change in pain over the entire study period depends only on the prevalence in the first wave and last wave and not on the prevalence in any of the 9 intermediate waves. If the first or last wave has unusually high or low prevalence, then conclusions about the net change across the study period could be skewed. With respect to missing data, a little over 6% of baseline observations are lost-to-follow-up. It is possible that missing observations disproportionately fall into one pain outcome. Although this is conceivable, it is also the case that supplementary analysis showed missing data to be not different from valid data in any compositional way. Finally, the study examined a relatively old population to address issues of expanding longevity among older-old Americans. It may be that trends among younger-aged cohorts differ, and it is also possible that selection into this older adult group affects findings.

In sum, this study concludes an expansion of pain morbidity for men and women aged 70 and 80 in the United States from 1993 to 2018, but no expansion for those aged 90. The results beg the question of why many older people are living more years with limiting pain. Clearly, more research is required, but increases in obesity (46), depression (47), and chronic diseases (48) may be part of the answer. Furthermore, other research has shown decreasing health expectancy levels among certain subsets of the population. Socioeconomic factors appear to play an important role as health expectancy has been found to be stagnating among lower-middle classes in the United States (49). Furthermore, Cantu et al. (50) report those with less a high school education are losing healthy life expectancy years due to both morbidity and disability relative to those more educated. In any case, for the population on balance, the results presented here are consistent with an expansion of morbidity hypothesis that suggests trade-offs between living longer lives and living healthy lives at very old ages.

## Supplementary Material

glae157_suppl_Supplementary_Material
