# Supplementary material for: Are We Adding Pain-Free Years to Life? A Test of Compression Versus Expansion of Morbidity
Source: J Gerontol A Biol Sci Med Sci. 2024 Jun 15;79(8):glae157. doi: 10.1093/gerona/glae157 (PMC11253204; doi:10.1093/gerona/glae157)
Supplement: glae157_suppl_Supplementary_Material [file glae157_suppl_supplementary_material.docx]

Supplementary materials

eTable 1: Number of observations by baseline wave and sex, p. 2

eTable 2: 95% confidence intervals for absolute and relative estimates, population and status-based, p. 3-6

eTable 3: Status based complete point estimates, p. 7-12

eTable 4: 95% confidence intervals for status based absolute and relative estimates, p. 13-24

eTable1: Number of observations by baseline wave and sex^1^

| Baseline wave | Follow-up wave | N males | N females | N missing |
| --- | --- | --- | --- | --- |
| 1993 | 1995 | 2,804 | 4,189 | 425 |
| 1998 | 2000 | 2,949 | 4,330 | 345 |
| 2000 | 2002 | 2,749 | 4,029 | 352 |
| 2002 | 2004 | 2,794 | 4,102 | 320 |
| 2004 | 2006 | 2,897 | 4,126 | 282 |
| 2006 | 2008 | 2,983 | 4,269 | 289 |
| 2008 | 2010 | 3,090 | 4,366 | 371 |
| 2010 | 2012 | 3,275 | 4,426 | 411 |
| 2012 | 2014 | 3,280 | 4,323 | 522 |
| 2014 | 2016 | 3,060 | 3,948 | 640 |
| 2016 | 2018 | 2,651 | 3,367 | 960 |
| Total |  | 32,532 | 45,475 | 4,917 |

^1^N’s provided for males and females are the number recording a valid baseline and follow-up observation. N’s for missing are the number with unknown status at follow-up.

eTable 2: 95% confidence intervals for absolute and relative estimates, population and status-based

| Absolute estimates | | | | | | | | | | | | | | |
| --- | --- | --- | --- | --- | --- | --- | --- | --- | --- | --- | --- | --- | --- | --- |
| Sex | Age | Est. | 1993 | | 1998 | | 2000 | | 2002 | | 2004 | | 2006 | |
|  |  |  | Lo | Hi | Lo | Hi | Lo | Hi | Lo | Hi | Lo | Hi | Lo | Hi |
| M | 70 | PFLE | 10.76 | 12.09 | 8.35 | 9.94 | 8.98 | 10.39 | 10.13 | 11.60 | 9.07 | 10.51 | 10.92 | 12.49 |
|  |  | NLPLE | 1.03 | 1.46 | 0.75 | 1.17 | 1.10 | 1.59 | 1.37 | 1.96 | 1.32 | 1.86 | 1.42 | 1.98 |
|  |  | LPLE | 1.20 | 1.70 | 1.58 | 2.51 | 1.67 | 2.32 | 1.94 | 2.76 | 2.50 | 3.42 | 2.08 | 2.80 |
|  |  | TLE | 13.40 | 14.89 | 11.30 | 12.90 | 12.28 | 13.69 | 14.07 | 15.79 | 13.52 | 15.05 | 14.98 | 16.60 |
|  | 80 | PFLE | 5.59 | 6.67 | 5.27 | 6.50 | 5.08 | 6.09 | 5.79 | 6.93 | 5.21 | 6.36 | 6.66 | 7.88 |
|  |  | NLPLE | 0.70 | 1.09 | 0.61 | 1.03 | 0.68 | 1.11 | 0.72 | 1.17 | 0.84 | 1.35 | 0.69 | 1.11 |
|  |  | LPLE | 0.87 | 1.34 | 1.05 | 1.78 | 1.16 | 1.75 | 1.30 | 2.04 | 1.60 | 2.42 | 1.03 | 1.61 |
|  |  | TLE | 7.55 | 8.68 | 7.35 | 8.69 | 7.33 | 8.43 | 8.26 | 9.66 | 8.17 | 9.45 | 8.87 | 10.22 |
|  | 90 | PFLE | 2.49 | 3.48 | 3.04 | 4.25 | 2.64 | 3.63 | 2.86 | 3.94 | 2.63 | 3.75 | 3.32 | 4.64 |
|  |  | NLPLE | 0.38 | 0.79 | 0.38 | 0.95 | 0.35 | 0.78 | 0.33 | 0.73 | 0.42 | 0.97 | 0.29 | 0.66 |
|  |  | LPLE | 0.53 | 1.07 | 0.54 | 1.31 | 0.64 | 1.25 | 0.75 | 1.56 | 0.88 | 1.79 | 0.50 | 1.00 |
|  |  | TLE | 3.81 | 4.82 | 4.38 | 6.02 | 4.00 | 5.20 | 4.37 | 5.65 | 4.42 | 5.81 | 4.43 | 5.90 |
| F | 70 | PFLE | 11.42 | 12.89 | 10.08 | 11.59 | 10.37 | 11.74 | 10.08 | 11.81 | 9.63 | 11.01 | 10.38 | 11.80 |
|  |  | NLPLE | 1.17 | 1.63 | 1.82 | 2.55 | 1.61 | 2.17 | 1.59 | 2.11 | 1.79 | 2.45 | 1.83 | 2.42 |
|  |  | LPLE | 2.77 | 3.57 | 3.30 | 4.21 | 3.45 | 4.36 | 3.81 | 4.76 | 4.28 | 5.37 | 3.98 | 4.90 |
|  |  | TLE | 15.88 | 17.65 | 16.03 | 17.53 | 16.03 | 17.60 | 16.28 | 18.08 | 16.56 | 18.09 | 16.93 | 18.44 |
|  | 80 | PFLE | 6.58 | 7.88 | 6.03 | 7.08 | 6.16 | 7.22 | 6.61 | 7.99 | 5.80 | 6.81 | 6.35 | 7.35 |
|  |  | NLPLE | 0.75 | 1.17 | 1.01 | 1.47 | 1.02 | 1.56 | 0.94 | 1.40 | 0.89 | 1.32 | 0.99 | 1.52 |
|  |  | LPLE | 2.08 | 2.80 | 2.22 | 2.88 | 2.05 | 2.75 | 2.19 | 2.98 | 2.60 | 3.43 | 2.44 | 3.17 |
|  |  | TLE | 9.83 | 11.46 | 9.74 | 10.98 | 9.71 | 10.98 | 10.26 | 11.88 | 9.79 | 11.18 | 10.21 | 11.58 |
|  | 90 | PFLE | 3.34 | 4.60 | 3.00 | 4.05 | 3.16 | 4.15 | 3.74 | 5.10 | 3.03 | 3.89 | 3.08 | 3.95 |
|  |  | NLPLE | 0.45 | 0.87 | 0.46 | 0.86 | 0.55 | 1.10 | 0.48 | 0.92 | 0.36 | 0.68 | 0.44 | 0.91 |
|  |  | LPLE | 1.38 | 2.15 | 1.30 | 2.00 | 1.07 | 1.72 | 1.09 | 1.83 | 1.29 | 2.07 | 1.29 | 2.03 |
|  |  | TLE | 5.59 | 7.13 | 5.25 | 6.55 | 5.16 | 6.41 | 5.74 | 7.39 | 5.05 | 6.26 | 5.22 | 6.49 |

Continued

eTable 2: Continued

| Absolute estimates | | | | | | | | | | | | |
| --- | --- | --- | --- | --- | --- | --- | --- | --- | --- | --- | --- | --- |
| Sex | Age | Est. | 2008 | | 2010 | | 2012 | | 2014 | | 2016 | |
|  |  |  | Lo | Hi | Lo | Hi | Lo | Hi | Lo | Hi | Lo | Hi |
| M | 70 | PFLE | 8.69 | 9.97 | 10.55 | 12.16 | 8.70 | 10.11 | 8.00 | 9.65 | 8.65 | 10.57 |
|  |  | NLPLE | 1.81 | 2.44 | 1.70 | 2.29 | 2.03 | 2.87 | 1.96 | 2.73 | 2.29 | 3.29 |
|  |  | LPLE | 2.30 | 3.17 | 2.53 | 3.40 | 2.63 | 3.46 | 2.35 | 3.24 | 2.95 | 4.13 |
|  |  | TLE | 13.50 | 14.81 | 15.52 | 17.16 | 14.07 | 15.54 | 13.09 | 14.69 | 15.00 | 16.76 |
|  | 80 | PFLE | 4.76 | 5.69 | 6.23 | 7.52 | 5.29 | 6.41 | 4.83 | 5.75 | 5.21 | 6.41 |
|  |  | NLPLE | 0.94 | 1.39 | 0.92 | 1.45 | 0.93 | 1.42 | 1.10 | 1.60 | 1.20 | 1.77 |
|  |  | LPLE | 1.35 | 2.05 | 1.51 | 2.28 | 1.40 | 2.01 | 1.22 | 1.71 | 1.46 | 2.15 |
|  |  | TLE | 7.59 | 8.61 | 9.25 | 10.63 | 8.17 | 9.25 | 7.59 | 8.54 | 8.38 | 9.66 |
|  | 90 | PFLE | 2.35 | 3.19 | 3.15 | 4.37 | 2.75 | 3.77 | 2.49 | 3.37 | 2.58 | 3.54 |
|  |  | NLPLE | 0.39 | 0.73 | 0.45 | 0.92 | 0.36 | 0.76 | 0.48 | 0.88 | 0.47 | 0.95 |
|  |  | LPLE | 0.65 | 1.22 | 0.77 | 1.54 | 0.60 | 1.08 | 0.54 | 0.95 | 0.55 | 1.05 |
|  |  | TLE | 3.81 | 4.73 | 4.80 | 6.22 | 4.14 | 5.21 | 3.85 | 4.77 | 3.92 | 5.17 |
| F | 70 | PFLE | 8.35 | 9.62 | 11.78 | 13.42 | 8.76 | 10.27 | 8.02 | 9.33 | 9.65 | 11.29 |
|  |  | NLPLE | 1.89 | 2.51 | 2.14 | 2.82 | 2.52 | 3.28 | 2.18 | 2.93 | 2.43 | 3.28 |
|  |  | LPLE | 4.49 | 5.44 | 4.13 | 5.06 | 5.58 | 6.88 | 4.92 | 6.05 | 4.69 | 5.88 |
|  |  | TLE | 15.45 | 16.76 | 18.84 | 20.65 | 17.68 | 19.46 | 15.83 | 17.42 | 17.66 | 19.44 |
|  | 80 | PFLE | 5.22 | 6.13 | 7.26 | 8.72 | 5.88 | 7.44 | 5.16 | 6.02 | 6.09 | 7.48 |
|  |  | NLPLE | 1.13 | 1.60 | 1.27 | 1.83 | 1.31 | 1.83 | 1.15 | 1.65 | 1.23 | 1.88 |
|  |  | LPLE | 2.36 | 3.06 | 2.54 | 3.44 | 3.20 | 4.34 | 2.80 | 3.50 | 2.58 | 3.39 |
|  |  | TLE | 9.18 | 10.33 | 11.67 | 13.44 | 11.05 | 12.65 | 9.54 | 10.67 | 10.51 | 11.96 |
|  | 90 | PFLE | 2.79 | 3.55 | 3.82 | 5.13 | 3.53 | 4.99 | 2.69 | 3.44 | 3.20 | 4.39 |
|  |  | NLPLE | 0.51 | 0.98 | 0.62 | 1.09 | 0.54 | 0.95 | 0.48 | 0.89 | 0.51 | 0.97 |
|  |  | LPLE | 1.08 | 1.73 | 1.38 | 2.23 | 1.57 | 2.57 | 1.39 | 2.04 | 1.19 | 1.85 |
|  |  | TLE | 4.76 | 5.74 | 6.27 | 7.94 | 6.13 | 7.82 | 4.94 | 5.89 | 5.34 | 6.75 |

Continued

eTable 2: Continued

| Relative estimates | | | | | | | | | | | | | | |
| --- | --- | --- | --- | --- | --- | --- | --- | --- | --- | --- | --- | --- | --- | --- |
| Sex | Age | Est. | 1993 | | 1998 | | 2000 | | 2002 | | 2004 | | 2006 | |
|  |  |  | Lo | Hi | Lo | Hi | Lo | Hi | Lo | Hi | Lo | Hi | Lo | Hi |
| M | 70 | PFLE | 76.16% | 85.54% | 68.91% | 82.05% | 69.16% | 80.07% | 68.14% | 78.05% | 63.33% | 73.42% | 69.30% | 79.33% |
|  |  | NLPLE | 7.32% | 10.33% | 6.17% | 9.66% | 8.50% | 12.23% | 9.20% | 13.18% | 9.25% | 13.01% | 9.05% | 12.59% |
|  |  | LPLE | 8.48% | 12.06% | 13.08% | 20.75% | 12.86% | 17.85% | 13.03% | 18.57% | 17.47% | 23.91% | 13.18% | 17.77% |
|  | 80 | PFLE | 69.44% | 82.92% | 66.08% | 81.43% | 64.56% | 77.41% | 64.66% | 77.45% | 59.51% | 72.65% | 70.81% | 83.70% |
|  |  | NLPLE | 8.68% | 13.50% | 7.65% | 12.94% | 8.67% | 14.06% | 8.01% | 13.13% | 9.60% | 15.44% | 7.30% | 11.81% |
|  |  | LPLE | 10.83% | 16.61% | 13.14% | 22.35% | 14.72% | 22.19% | 14.47% | 22.83% | 18.26% | 27.61% | 10.93% | 17.11% |
|  | 90 | PFLE | 58.92% | 82.55% | 59.87% | 83.86% | 58.13% | 79.88% | 57.12% | 78.75% | 52.40% | 74.79% | 66.00% | 92.24% |
|  |  | NLPLE | 9.05% | 18.77% | 7.57% | 18.67% | 7.76% | 17.25% | 6.59% | 14.53% | 8.45% | 19.25% | 5.86% | 13.16% |
|  |  | LPLE | 12.48% | 25.41% | 10.73% | 25.81% | 14.09% | 27.62% | 14.96% | 31.26% | 17.55% | 35.70% | 9.90% | 19.79% |
| F | 70 | PFLE | 68.62% | 77.46% | 60.26% | 69.26% | 61.67% | 69.80% | 58.93% | 69.06% | 55.87% | 63.91% | 58.78% | 66.81% |
|  |  | NLPLE | 7.00% | 9.80% | 10.89% | 15.22% | 9.59% | 12.92% | 9.27% | 12.33% | 10.38% | 14.21% | 10.34% | 13.69% |
|  |  | LPLE | 16.62% | 21.48% | 19.73% | 25.19% | 20.53% | 25.92% | 22.30% | 27.86% | 24.84% | 31.16% | 22.55% | 27.75% |
|  | 80 | PFLE | 62.24% | 74.58% | 58.50% | 68.64% | 59.86% | 70.16% | 60.21% | 72.81% | 55.90% | 65.65% | 58.86% | 68.15% |
|  |  | NLPLE | 7.13% | 11.09% | 9.78% | 14.29% | 9.89% | 15.12% | 8.58% | 12.74% | 8.59% | 12.72% | 9.19% | 14.13% |
|  |  | LPLE | 19.69% | 26.50% | 21.56% | 27.94% | 19.91% | 26.73% | 19.95% | 27.15% | 25.03% | 33.07% | 22.60% | 29.41% |
|  | 90 | PFLE | 53.34% | 73.53% | 51.95% | 70.00% | 55.62% | 72.97% | 57.56% | 78.64% | 54.28% | 69.72% | 54.12% | 69.33% |
|  |  | NLPLE | 7.12% | 13.85% | 7.94% | 14.86% | 9.64% | 19.29% | 7.46% | 14.10% | 6.54% | 12.15% | 7.73% | 16.06% |
|  |  | LPLE | 21.97% | 34.38% | 22.44% | 34.62% | 18.83% | 30.22% | 16.84% | 28.23% | 23.21% | 37.07% | 22.74% | 35.65% |

Continued

eTable 2: Continued

| Relative estimates | | | | | | | | | | | | |
| --- | --- | --- | --- | --- | --- | --- | --- | --- | --- | --- | --- | --- |
| Sex | Age | Est. | 2008 | | 2010 | | 2012 | | 2014 | | 2016 | |
|  |  |  | Lo | Hi | Lo | Hi | Lo | Hi | Lo | Hi | Lo | Hi |
| M | 70 | PFLE | 61.52% | 70.62% | 64.82% | 74.67% | 58.77% | 68.27% | 57.76% | 69.66% | 54.51% | 66.58% |
|  |  | NLPLE | 12.79% | 17.27% | 10.45% | 14.06% | 13.73% | 19.41% | 14.16% | 19.69% | 14.46% | 20.70% |
|  |  | LPLE | 16.28% | 22.49% | 15.53% | 20.86% | 17.75% | 23.39% | 16.96% | 23.37% | 18.58% | 26.02% |
|  | 80 | PFLE | 59.25% | 70.90% | 62.97% | 75.95% | 60.82% | 73.71% | 60.20% | 71.70% | 58.12% | 71.49% |
|  |  | NLPLE | 11.77% | 17.35% | 9.29% | 14.60% | 10.70% | 16.29% | 13.76% | 19.90% | 13.43% | 19.73% |
|  |  | LPLE | 16.85% | 25.55% | 15.24% | 23.06% | 16.14% | 23.10% | 15.20% | 21.36% | 16.27% | 23.95% |
|  | 90 | PFLE | 56.61% | 76.75% | 57.94% | 80.49% | 59.89% | 82.04% | 59.06% | 80.15% | 58.58% | 80.51% |
|  |  | NLPLE | 9.46% | 17.50% | 8.31% | 16.88% | 7.86% | 16.45% | 11.33% | 20.87% | 10.71% | 21.59% |
|  |  | LPLE | 15.72% | 29.21% | 14.24% | 28.42% | 12.99% | 23.61% | 12.79% | 22.48% | 12.61% | 23.84% |
| F | 70 | PFLE | 51.86% | 59.73% | 59.99% | 68.35% | 47.15% | 55.30% | 48.17% | 56.06% | 52.41% | 61.29% |
|  |  | NLPLE | 11.74% | 15.61% | 10.88% | 14.35% | 13.55% | 17.69% | 13.07% | 17.61% | 13.18% | 17.78% |
|  |  | LPLE | 27.88% | 33.77% | 21.04% | 25.76% | 30.05% | 37.06% | 29.55% | 36.31% | 25.44% | 31.91% |
|  | 80 | PFLE | 53.78% | 63.15% | 58.42% | 70.12% | 49.70% | 62.87% | 51.10% | 59.68% | 54.45% | 66.84% |
|  |  | NLPLE | 11.61% | 16.48% | 10.23% | 14.69% | 11.07% | 15.43% | 11.38% | 16.37% | 10.98% | 16.78% |
|  |  | LPLE | 24.32% | 31.55% | 20.43% | 27.70% | 27.05% | 36.71% | 27.78% | 34.71% | 23.08% | 30.27% |
|  | 90 | PFLE | 53.67% | 68.35% | 54.46% | 73.22% | 51.30% | 72.60% | 50.17% | 64.05% | 53.89% | 73.99% |
|  |  | NLPLE | 9.85% | 18.77% | 8.81% | 15.59% | 7.85% | 13.76% | 8.93% | 16.60% | 8.65% | 16.30% |
|  |  | LPLE | 20.83% | 33.18% | 19.69% | 31.78% | 22.84% | 37.35% | 25.92% | 38.01% | 20.05% | 31.21% |

eTable 3: Status based complete point estimates

| Absolute estimates | | | | | | | | | | | | | | | |
| --- | --- | --- | --- | --- | --- | --- | --- | --- | --- | --- | --- | --- | --- | --- | --- |
| Baseline status = No pain | | | | | | | | | | | | | | | |
|  |  |  | 1993 | 1998 | 2000 | 2002 | 2004 | 2006 | 2008 | 2010 | 2012 | 2014 | 2016 | β | p-value |
| M | 70 | PFLE | 12.24 | 10.07 | 10.48 | 11.84 | 11.04 | 12.99 | 10.58 | 12.90 | 11.03 | 10.38 | 11.31 | -0.006 | 0.910 |
|  |  | NLPLE | 0.98 | 0.76 | 1.08 | 1.40 | 1.33 | 1.36 | 1.67 | 1.55 | 1.87 | 1.87 | 2.09 | 0.054 | 0.000 |
|  |  | LPLE | 1.09 | 1.42 | 1.48 | 1.79 | 2.25 | 1.70 | 2.07 | 2.19 | 2.20 | 1.96 | 2.55 | 0.053 | 0.000 |
|  |  | TLE | 14.31 | 12.25 | 13.03 | 15.03 | 14.62 | 16.05 | 14.32 | 16.64 | 15.09 | 14.21 | 15.95 | 0.101 | 0.075 |
|  | 80 | PFLE | 6.95 | 6.73 | 6.56 | 7.33 | 6.91 | 8.23 | 6.37 | 8.08 | 7.21 | 6.61 | 7.09 | 0.013 | 0.649 |
|  |  | NLPLE | 0.61 | 0.57 | 0.62 | 0.70 | 0.78 | 0.61 | 0.79 | 0.85 | 0.73 | 0.90 | 0.95 | 0.015 | 0.001 |
|  |  | LPLE | 0.69 | 0.83 | 0.88 | 1.07 | 1.29 | 0.78 | 1.07 | 1.19 | 0.98 | 0.80 | 1.03 | 0.009 | 0.292 |
|  |  | TLE | 8.25 | 8.14 | 8.07 | 9.10 | 8.98 | 9.62 | 8.23 | 10.12 | 8.93 | 8.31 | 9.06 | 0.037 | 0.229 |
|  | 90 | PFLE | 3.69 | 4.44 | 4.11 | 4.27 | 4.11 | 4.58 | 3.57 | 4.54 | 4.17 | 3.82 | 3.88 | -0.003 | 0.858 |
|  |  | NLPLE | 0.31 | 0.37 | 0.30 | 0.28 | 0.38 | 0.21 | 0.28 | 0.38 | 0.22 | 0.32 | 0.32 | -0.001 | 0.658 |
|  |  | LPLE | 0.35 | 0.44 | 0.43 | 0.53 | 0.62 | 0.30 | 0.41 | 0.50 | 0.32 | 0.24 | 0.27 | -0.007 | 0.223 |
|  |  | TLE | 4.35 | 5.24 | 4.84 | 5.08 | 5.11 | 5.09 | 4.27 | 5.43 | 4.70 | 4.38 | 4.47 | -0.011 | 0.578 |
| F | 70 | PFLE | 13.25 | 12.24 | 12.50 | 12.52 | 11.90 | 12.91 | 10.84 | 14.45 | 11.50 | 11.04 | 12.47 | -0.040 | 0.402 |
|  |  | NLPLE | 1.14 | 1.77 | 1.66 | 1.57 | 1.70 | 1.78 | 1.88 | 2.09 | 2.52 | 2.09 | 2.36 | 0.048 | 0.000 |
|  |  | LPLE | 2.39 | 2.99 | 2.91 | 3.31 | 3.71 | 3.50 | 3.68 | 3.41 | 4.77 | 3.98 | 3.82 | 0.073 | 0.001 |
|  |  | TLE | 16.77 | 17.00 | 17.07 | 17.40 | 17.31 | 18.19 | 16.40 | 19.96 | 18.79 | 17.11 | 18.65 | 0.081 | 0.087 |
|  | 80 | PFLE | 8.43 | 7.84 | 7.94 | 8.61 | 7.64 | 8.18 | 7.18 | 9.62 | 8.36 | 7.39 | 8.56 | 0.005 | 0.882 |
|  |  | NLPLE | 0.68 | 0.93 | 0.97 | 0.90 | 0.81 | 0.91 | 1.03 | 1.14 | 1.20 | 0.97 | 1.13 | 0.017 | 0.005 |
|  |  | LPLE | 1.63 | 1.76 | 1.59 | 1.76 | 2.04 | 1.92 | 1.69 | 1.93 | 2.52 | 1.95 | 1.77 | 0.018 | 0.117 |
|  |  | TLE | 10.74 | 10.53 | 10.50 | 11.28 | 10.48 | 11.01 | 9.90 | 12.69 | 12.08 | 10.30 | 11.47 | 0.040 | 0.303 |
|  | 90 | PFLE | 5.07 | 4.57 | 4.60 | 5.45 | 4.45 | 4.35 | 4.22 | 5.74 | 5.58 | 4.22 | 5.10 | 0.007 | 0.807 |
|  |  | NLPLE | 0.34 | 0.39 | 0.44 | 0.44 | 0.31 | 0.34 | 0.42 | 0.49 | 0.43 | 0.31 | 0.42 | 0.001 | 0.631 |
|  |  | LPLE | 0.96 | 0.87 | 0.72 | 0.78 | 0.86 | 0.79 | 0.59 | 0.85 | 1.12 | 0.74 | 0.58 | -0.006 | 0.406 |
|  |  | TLE | 6.37 | 5.82 | 5.76 | 6.68 | 5.61 | 5.48 | 5.23 | 7.07 | 7.13 | 5.27 | 6.09 | 0.002 | 0.954 |

Continued

eTable 3: Continued

| Absolute estimates | | | | | | | | | | | | | | | |
| --- | --- | --- | --- | --- | --- | --- | --- | --- | --- | --- | --- | --- | --- | --- | --- |
| Baseline status = Non-limiting pain | | | | | | | | | | | | | | | |
|  |  |  | 1993 | 1998 | 2000 | 2002 | 2004 | 2006 | 2008 | 2010 | 2012 | 2014 | 2016 | β | p-value |
| M | 70 | PFLE | 9.42 | 7.45 | 7.17 | 8.59 | 7.71 | 9.31 | 7.27 | 8.73 | 6.65 | 6.69 | 7.54 | -0.065 | 0.147 |
|  |  | NLPLE | 3.27 | 3.12 | 3.70 | 4.00 | 3.72 | 3.83 | 4.48 | 4.20 | 5.17 | 4.54 | 5.38 | 0.091 | 0.000 |
|  |  | LPLE | 1.39 | 1.80 | 2.29 | 2.13 | 2.85 | 2.39 | 2.36 | 2.88 | 2.68 | 2.76 | 3.03 | 0.062 | 0.000 |
|  |  | TLE | 14.09 | 12.36 | 13.17 | 14.72 | 14.28 | 15.54 | 14.11 | 15.81 | 14.51 | 13.99 | 15.95 | 0.088 | 0.063 |
|  | 80 | PFLE | 4.00 | 3.90 | 3.51 | 4.27 | 3.60 | 5.25 | 3.06 | 4.79 | 3.60 | 3.18 | 3.81 | -0.013 | 0.688 |
|  |  | NLPLE | 2.92 | 2.92 | 3.01 | 3.12 | 3.29 | 3.03 | 3.41 | 3.30 | 3.61 | 3.56 | 3.72 | 0.037 | 0.000 |
|  |  | LPLE | 1.07 | 1.09 | 1.40 | 1.44 | 1.81 | 1.12 | 1.43 | 1.70 | 1.43 | 1.36 | 1.56 | 0.017 | 0.117 |
|  |  | TLE | 7.99 | 7.91 | 7.92 | 8.84 | 8.71 | 9.40 | 7.90 | 9.79 | 8.64 | 8.10 | 9.09 | 0.041 | 0.177 |
|  | 90 | PFLE | 1.23 | 1.64 | 1.50 | 1.69 | 1.27 | 2.38 | 0.89 | 2.25 | 1.63 | 1.08 | 1.43 | 0.001 | 0.949 |
|  |  | NLPLE | 2.30 | 2.43 | 2.29 | 2.34 | 2.64 | 2.35 | 2.38 | 2.60 | 2.54 | 2.50 | 2.45 | 0.009 | 0.093 |
|  |  | LPLE | 0.68 | 0.54 | 0.61 | 0.84 | 0.98 | 0.43 | 0.68 | 0.89 | 0.66 | 0.48 | 0.63 | -0.002 | 0.780 |
|  |  | TLE | 4.21 | 4.61 | 4.40 | 4.87 | 4.89 | 5.17 | 3.95 | 5.74 | 4.84 | 4.06 | 4.51 | 0.008 | 0.746 |
| F | 70 | PFLE | 10.18 | 7.95 | 9.14 | 8.66 | 8.66 | 9.47 | 7.04 | 10.90 | 7.79 | 6.88 | 9.10 | -0.051 | 0.379 |
|  |  | NLPLE | 3.37 | 4.82 | 3.90 | 4.01 | 4.62 | 4.32 | 4.38 | 4.78 | 5.11 | 4.88 | 5.10 | 0.061 | 0.004 |
|  |  | LPLE | 3.15 | 3.77 | 3.69 | 3.94 | 4.22 | 3.92 | 4.66 | 4.04 | 5.88 | 5.12 | 4.33 | 0.079 | 0.007 |
|  |  | TLE | 16.70 | 16.53 | 16.74 | 16.62 | 17.50 | 17.71 | 16.09 | 19.72 | 18.78 | 16.88 | 18.53 | 0.089 | 0.076 |
|  | 80 | PFLE | 5.61 | 4.38 | 5.04 | 5.36 | 4.64 | 5.14 | 4.02 | 6.05 | 4.81 | 4.16 | 5.05 | -0.018 | 0.531 |
|  |  | NLPLE | 2.91 | 3.48 | 3.37 | 3.16 | 3.31 | 3.39 | 3.56 | 3.75 | 3.83 | 3.69 | 3.72 | 0.034 | 0.001 |
|  |  | LPLE | 2.32 | 2.30 | 2.00 | 2.09 | 2.79 | 2.42 | 2.07 | 2.65 | 3.24 | 2.59 | 2.47 | 0.025 | 0.133 |
|  |  | TLE | 10.83 | 10.16 | 10.41 | 10.60 | 10.75 | 10.95 | 9.66 | 12.45 | 11.88 | 10.44 | 11.24 | 0.040 | 0.268 |
|  | 90 | PFLE | 2.74 | 1.98 | 2.30 | 2.90 | 2.00 | 2.17 | 1.74 | 2.60 | 2.20 | 1.90 | 2.09 | 2.74 | 0.197 |
|  |  | NLPLE | 2.48 | 2.44 | 2.83 | 2.43 | 2.33 | 2.64 | 2.56 | 2.84 | 2.69 | 2.63 | 2.63 | 2.48 | 0.226 |
|  |  | LPLE | 1.56 | 1.19 | 0.91 | 0.96 | 1.72 | 1.40 | 0.66 | 1.59 | 1.39 | 1.03 | 1.29 | 1.56 | 0.843 |
|  |  | TLE | 6.78 | 5.61 | 6.04 | 6.30 | 6.06 | 6.21 | 4.96 | 7.03 | 6.28 | 5.56 | 6.01 | 6.78 | 0.562 |

Continued

eTable 3: Continued

| Absolute estimates | | | | | | | | | | | | | | | |
| --- | --- | --- | --- | --- | --- | --- | --- | --- | --- | --- | --- | --- | --- | --- | --- |
| Baseline status = Limiting pain | | | | | | | | | | | | | | | |
|  |  |  | 1993 | 1998 | 2000 | 2002 | 2004 | 2006 | 2008 | 2010 | 2012 | 2014 | 2016 | β | p-value |
| M | 70 | PFLE | 7.58 | 5.30 | 6.42 | 7.10 | 5.53 | 7.82 | 5.64 | 7.65 | 6.14 | 5.36 | 6.68 | -0.022 | 0.628 |
|  |  | NLPLE | 1.35 | 0.67 | 1.16 | 1.34 | 1.21 | 1.48 | 1.99 | 1.80 | 2.05 | 1.90 | 2.51 | 0.061 | 0.001 |
|  |  | LPLE | 3.86 | 5.17 | 4.90 | 5.41 | 6.13 | 5.28 | 5.61 | 5.82 | 5.93 | 5.37 | 6.41 | 0.076 | 0.004 |
|  |  | TLE | 12.80 | 11.14 | 12.48 | 13.86 | 12.88 | 14.58 | 13.24 | 15.27 | 14.11 | 12.63 | 15.60 | 0.114 | 0.043 |
|  | 80 | PFLE | 3.09 | 2.64 | 2.08 | 3.22 | 2.56 | 4.12 | 2.12 | 3.68 | 2.62 | 2.15 | 2.87 | -0.005 | 0.873 |
|  |  | NLPLE | 0.68 | 0.53 | 0.66 | 0.74 | 0.74 | 0.70 | 0.85 | 0.90 | 0.92 | 0.94 | 1.19 | 0.022 | 0.000 |
|  |  | LPLE | 3.23 | 4.03 | 4.12 | 4.31 | 4.63 | 3.70 | 4.36 | 4.56 | 4.32 | 3.86 | 4.43 | 0.030 | 0.111 |
|  |  | TLE | 6.99 | 7.21 | 6.86 | 8.28 | 7.94 | 8.52 | 7.33 | 9.14 | 7.86 | 6.96 | 8.50 | 0.047 | 0.184 |
|  | 90 | PFLE | 1.04 | 1.16 | 0.47 | 1.19 | 1.04 | 1.82 | 0.62 | 1.42 | 0.79 | 0.65 | 0.83 | -0.009 | 0.646 |
|  |  | NLPLE | 0.24 | 0.41 | 0.30 | 0.38 | 0.38 | 0.28 | 0.25 | 0.38 | 0.33 | 0.35 | 0.42 | 0.003 | 0.292 |
|  |  | LPLE | 2.45 | 3.03 | 2.78 | 3.23 | 3.39 | 2.59 | 3.03 | 3.45 | 2.88 | 2.68 | 2.80 | 0.006 | 0.714 |
|  |  | TLE | 3.74 | 4.61 | 3.55 | 4.79 | 4.80 | 4.69 | 3.91 | 5.26 | 4.01 | 3.68 | 4.05 | 0.000 | 0.996 |
| F | 70 | PFLE | 8.69 | 7.10 | 7.47 | 7.64 | 6.95 | 7.29 | 6.15 | 9.34 | 6.55 | 5.60 | 7.29 | -0.060 | 0.215 |
|  |  | NLPLE | 1.24 | 1.94 | 1.61 | 1.60 | 1.77 | 1.74 | 1.82 | 2.13 | 2.29 | 1.92 | 2.32 | 0.037 | 0.002 |
|  |  | LPLE | 6.11 | 6.74 | 6.98 | 7.24 | 8.13 | 7.20 | 7.51 | 7.36 | 9.16 | 8.21 | 8.34 | 0.100 | 0.001 |
|  |  | TLE | 16.04 | 15.78 | 16.07 | 16.48 | 16.85 | 16.22 | 15.48 | 18.83 | 18.01 | 15.73 | 17.95 | 0.077 | 0.122 |
|  | 80 | PFLE | 4.24 | 3.63 | 3.73 | 4.29 | 3.50 | 3.93 | 3.11 | 5.01 | 3.68 | 2.97 | 3.87 | -0.017 | 0.538 |
|  |  | NLPLE | 0.80 | 1.01 | 1.03 | 0.91 | 0.80 | 0.98 | 1.01 | 1.28 | 1.15 | 1.01 | 1.18 | 0.014 | 0.025 |
|  |  | LPLE | 4.86 | 5.06 | 4.82 | 5.14 | 5.65 | 5.19 | 5.15 | 5.50 | 6.45 | 5.54 | 5.57 | 0.044 | 0.019 |
|  |  | TLE | 9.91 | 9.70 | 9.58 | 10.34 | 9.95 | 10.10 | 9.27 | 11.79 | 11.27 | 9.52 | 10.62 | 0.042 | 0.245 |
|  | 90 | PFLE | 1.69 | 1.55 | 1.49 | 1.98 | 1.39 | 1.74 | 1.23 | 2.16 | 1.70 | 1.27 | 1.59 | -0.004 | 0.786 |
|  |  | NLPLE | 0.49 | 0.48 | 0.61 | 0.47 | 0.29 | 0.53 | 0.47 | 0.69 | 0.48 | 0.46 | 0.49 | 0.001 | 0.866 |
|  |  | LPLE | 3.60 | 3.74 | 3.21 | 3.61 | 3.63 | 3.74 | 3.55 | 3.98 | 4.38 | 3.75 | 3.47 | 0.015 | 0.292 |
|  |  | TLE | 5.78 | 5.76 | 5.31 | 6.06 | 5.31 | 6.00 | 5.25 | 6.84 | 6.56 | 5.48 | 5.55 | 0.012 | 0.637 |

Continued

eTable 3: Contined

| Relative estimates | | | | | | | | | | | | | | | |
| --- | --- | --- | --- | --- | --- | --- | --- | --- | --- | --- | --- | --- | --- | --- | --- |
| Baseline status = No pain | | | | | | | | | | | | | | | |
|  |  |  | 1993 | 1998 | 2000 | 2002 | 2004 | 2006 | 2008 | 2010 | 2012 | 2014 | 2016 | β | p-value |
| M | 70 | PFLE | 0.855 | 0.822 | 0.804 | 0.788 | 0.755 | 0.809 | 0.739 | 0.775 | 0.731 | 0.731 | 0.709 | -0.034 | 0.000 |
|  |  | NLPLE | 0.069 | 0.062 | 0.083 | 0.093 | 0.091 | 0.085 | 0.116 | 0.093 | 0.124 | 0.132 | 0.131 | 0.036 | 0.000 |
|  |  | LPLE | 0.076 | 0.116 | 0.113 | 0.119 | 0.154 | 0.106 | 0.145 | 0.131 | 0.145 | 0.138 | 0.160 | 0.025 | 0.000 |
|  | 80 | PFLE | 0.842 | 0.827 | 0.814 | 0.806 | 0.770 | 0.856 | 0.774 | 0.798 | 0.808 | 0.796 | 0.782 | -0.013 | 0.000 |
|  |  | NLPLE | 0.074 | 0.071 | 0.077 | 0.077 | 0.087 | 0.063 | 0.096 | 0.084 | 0.082 | 0.108 | 0.105 | 0.018 | 0.000 |
|  |  | LPLE | 0.083 | 0.102 | 0.109 | 0.118 | 0.143 | 0.081 | 0.131 | 0.118 | 0.110 | 0.096 | 0.113 | 0.006 | 0.331 |
|  | 90 | PFLE | 0.849 | 0.846 | 0.849 | 0.840 | 0.804 | 0.900 | 0.837 | 0.837 | 0.886 | 0.872 | 0.867 | 0.011 | 0.016 |
|  |  | NLPLE | 0.072 | 0.070 | 0.061 | 0.056 | 0.075 | 0.041 | 0.066 | 0.070 | 0.046 | 0.073 | 0.072 | -0.002 | 0.782 |
|  |  | LPLE | 0.080 | 0.083 | 0.090 | 0.104 | 0.121 | 0.060 | 0.096 | 0.093 | 0.067 | 0.056 | 0.061 | -0.016 | 0.069 |
| F | 70 | PFLE | 0.790 | 0.720 | 0.732 | 0.720 | 0.688 | 0.710 | 0.661 | 0.724 | 0.612 | 0.645 | 0.669 | -0.027 | 0.000 |
|  |  | NLPLE | 0.068 | 0.104 | 0.097 | 0.090 | 0.098 | 0.098 | 0.114 | 0.105 | 0.134 | 0.122 | 0.126 | 0.025 | 0.000 |
|  |  | LPLE | 0.142 | 0.176 | 0.170 | 0.190 | 0.214 | 0.193 | 0.224 | 0.171 | 0.254 | 0.232 | 0.205 | 0.021 | 0.000 |
|  | 80 | PFLE | 0.785 | 0.744 | 0.756 | 0.764 | 0.729 | 0.743 | 0.726 | 0.758 | 0.692 | 0.717 | 0.747 | -0.012 | 0.023 |
|  |  | NLPLE | 0.063 | 0.088 | 0.092 | 0.080 | 0.077 | 0.083 | 0.104 | 0.090 | 0.099 | 0.094 | 0.099 | 0.015 | 0.002 |
|  |  | LPLE | 0.152 | 0.167 | 0.152 | 0.156 | 0.194 | 0.174 | 0.171 | 0.152 | 0.209 | 0.189 | 0.154 | 0.008 | 0.714 |
|  | 90 | PFLE | 0.796 | 0.785 | 0.798 | 0.817 | 0.793 | 0.793 | 0.806 | 0.811 | 0.782 | 0.800 | 0.836 | 0.005 | 0.217 |
|  |  | NLPLE | 0.054 | 0.066 | 0.076 | 0.066 | 0.054 | 0.062 | 0.081 | 0.069 | 0.060 | 0.059 | 0.068 | 0.003 | 0.568 |
|  |  | LPLE | 0.150 | 0.149 | 0.126 | 0.117 | 0.153 | 0.145 | 0.112 | 0.119 | 0.157 | 0.141 | 0.095 | -0.009 | 0.198 |

Continued

eTable 3: Continued

| Relative estimates | | | | | | | | | | | | | | | |
| --- | --- | --- | --- | --- | --- | --- | --- | --- | --- | --- | --- | --- | --- | --- | --- |
| Baseline status = Non-limiting pain | | | | | | | | | | | | | | | |
|  |  |  | 1993 | 1998 | 2000 | 2002 | 2004 | 2006 | 2008 | 2010 | 2012 | 2014 | 2016 | β | p-value |
| M | 70 | PFLE | 0.669 | 0.602 | 0.545 | 0.584 | 0.540 | 0.599 | 0.515 | 0.552 | 0.458 | 0.478 | 0.473 | -0.032 | 0.000 |
|  |  | NLPLE | 0.232 | 0.252 | 0.281 | 0.271 | 0.261 | 0.247 | 0.317 | 0.265 | 0.357 | 0.325 | 0.337 | 0.023 | 0.000 |
|  |  | LPLE | 0.099 | 0.146 | 0.174 | 0.145 | 0.199 | 0.154 | 0.167 | 0.182 | 0.185 | 0.197 | 0.190 | 0.024 | 0.000 |
|  | 80 | PFLE | 0.501 | 0.493 | 0.444 | 0.484 | 0.414 | 0.558 | 0.387 | 0.490 | 0.417 | 0.393 | 0.419 | -0.015 | 0.000 |
|  |  | NLPLE | 0.365 | 0.369 | 0.380 | 0.353 | 0.378 | 0.322 | 0.432 | 0.337 | 0.417 | 0.439 | 0.410 | 0.011 | 0.007 |
|  |  | LPLE | 0.134 | 0.138 | 0.176 | 0.163 | 0.208 | 0.120 | 0.181 | 0.173 | 0.166 | 0.168 | 0.171 | 0.009 | 0.037 |
|  | 90 | PFLE | 0.292 | 0.357 | 0.342 | 0.348 | 0.260 | 0.461 | 0.226 | 0.392 | 0.338 | 0.265 | 0.318 | -0.002 | 0.755 |
|  |  | NLPLE | 0.546 | 0.527 | 0.520 | 0.479 | 0.540 | 0.456 | 0.601 | 0.453 | 0.526 | 0.616 | 0.543 | 0.005 | 0.460 |
|  |  | LPLE | 0.162 | 0.117 | 0.139 | 0.173 | 0.201 | 0.083 | 0.172 | 0.155 | 0.136 | 0.118 | 0.139 | -0.007 | 0.279 |
| F | 70 | PFLE | 0.609 | 0.481 | 0.546 | 0.521 | 0.495 | 0.535 | 0.438 | 0.553 | 0.415 | 0.408 | 0.491 | -0.023 | 0.009 |
|  |  | NLPLE | 0.202 | 0.292 | 0.233 | 0.241 | 0.264 | 0.244 | 0.272 | 0.242 | 0.272 | 0.289 | 0.275 | 0.012 | 0.061 |
|  |  | LPLE | 0.189 | 0.228 | 0.221 | 0.237 | 0.241 | 0.221 | 0.290 | 0.205 | 0.313 | 0.303 | 0.234 | 0.019 | 0.012 |
|  | 80 | PFLE | 0.518 | 0.431 | 0.485 | 0.505 | 0.432 | 0.469 | 0.417 | 0.486 | 0.405 | 0.398 | 0.449 | -0.014 | 0.015 |
|  |  | NLPLE | 0.268 | 0.342 | 0.323 | 0.298 | 0.308 | 0.309 | 0.369 | 0.301 | 0.323 | 0.353 | 0.331 | 0.009 | 0.068 |
|  |  | LPLE | 0.214 | 0.226 | 0.192 | 0.197 | 0.260 | 0.221 | 0.214 | 0.213 | 0.273 | 0.248 | 0.220 | 0.008 | 0.088 |
|  | 90 | PFLE | 0.404 | 0.353 | 0.381 | 0.461 | 0.330 | 0.350 | 0.351 | 0.370 | 0.350 | 0.341 | 0.347 | -0.011 | 0.004 |
|  |  | NLPLE | 0.367 | 0.434 | 0.469 | 0.386 | 0.385 | 0.424 | 0.516 | 0.404 | 0.428 | 0.473 | 0.438 | 0.010 | 0.064 |
|  |  | LPLE | 0.229 | 0.213 | 0.151 | 0.153 | 0.285 | 0.226 | 0.132 | 0.226 | 0.222 | 0.185 | 0.215 | 0.000 | 0.964 |

Continued

eTable 3: Continued

| Relative estimates | | | | | | | | | | | | | | | |
| --- | --- | --- | --- | --- | --- | --- | --- | --- | --- | --- | --- | --- | --- | --- | --- |
| Baseline status = Limiting pain | | | | | | | | | | | | | | | |
|  |  |  | 1993 | 1998 | 2000 | 2002 | 2004 | 2006 | 2008 | 2010 | 2012 | 2014 | 2016 | β | p-value |
| M | 70 | PFLE | 0.592 | 0.476 | 0.514 | 0.513 | 0.430 | 0.536 | 0.426 | 0.501 | 0.435 | 0.424 | 0.428 | -0.023 | 0.000 |
|  |  | NLPLE | 0.106 | 0.060 | 0.093 | 0.097 | 0.094 | 0.102 | 0.151 | 0.118 | 0.145 | 0.151 | 0.161 | 0.036 | 0.001 |
|  |  | LPLE | 0.302 | 0.464 | 0.393 | 0.391 | 0.476 | 0.362 | 0.424 | 0.381 | 0.420 | 0.425 | 0.411 | 0.009 | 0.364 |
|  | 80 | PFLE | 0.441 | 0.366 | 0.303 | 0.389 | 0.323 | 0.484 | 0.289 | 0.402 | 0.334 | 0.310 | 0.338 | -0.013 | 0.058 |
|  |  | NLPLE | 0.097 | 0.074 | 0.097 | 0.090 | 0.093 | 0.082 | 0.116 | 0.099 | 0.117 | 0.135 | 0.141 | 0.025 | 0.001 |
|  |  | LPLE | 0.462 | 0.560 | 0.601 | 0.521 | 0.583 | 0.434 | 0.595 | 0.499 | 0.549 | 0.555 | 0.521 | 0.003 | 0.698 |
|  | 90 | PFLE | 0.279 | 0.252 | 0.133 | 0.248 | 0.216 | 0.388 | 0.160 | 0.271 | 0.198 | 0.176 | 0.205 | -0.013 | 0.165 |
|  |  | NLPLE | 0.066 | 0.090 | 0.083 | 0.079 | 0.079 | 0.059 | 0.065 | 0.073 | 0.084 | 0.096 | 0.104 | 0.012 | 0.084 |
|  |  | LPLE | 0.655 | 0.658 | 0.783 | 0.673 | 0.705 | 0.553 | 0.775 | 0.656 | 0.718 | 0.728 | 0.692 | 0.007 | 0.359 |
| F | 70 | PFLE | 0.542 | 0.450 | 0.465 | 0.463 | 0.412 | 0.449 | 0.397 | 0.496 | 0.364 | 0.356 | 0.406 | -0.024 | 0.000 |
|  |  | NLPLE | 0.077 | 0.123 | 0.100 | 0.097 | 0.105 | 0.107 | 0.118 | 0.113 | 0.127 | 0.122 | 0.129 | 0.018 | 0.004 |
|  |  | LPLE | 0.381 | 0.427 | 0.435 | 0.439 | 0.483 | 0.444 | 0.485 | 0.391 | 0.509 | 0.522 | 0.464 | 0.016 | 0.002 |
|  | 80 | PFLE | 0.428 | 0.374 | 0.389 | 0.414 | 0.352 | 0.389 | 0.335 | 0.425 | 0.326 | 0.312 | 0.364 | -0.014 | 0.006 |
|  |  | NLPLE | 0.081 | 0.104 | 0.108 | 0.088 | 0.080 | 0.097 | 0.109 | 0.109 | 0.102 | 0.106 | 0.111 | 0.011 | 0.007 |
|  |  | LPLE | 0.491 | 0.522 | 0.503 | 0.498 | 0.568 | 0.514 | 0.556 | 0.466 | 0.572 | 0.582 | 0.524 | 0.009 | 0.048 |
|  | 90 | PFLE | 0.292 | 0.269 | 0.281 | 0.327 | 0.262 | 0.289 | 0.234 | 0.316 | 0.259 | 0.232 | 0.286 | 0.005 | 0.210 |
|  |  | NLPLE | 0.084 | 0.083 | 0.114 | 0.078 | 0.055 | 0.088 | 0.090 | 0.101 | 0.073 | 0.084 | 0.088 | -0.001 | 0.895 |
|  |  | LPLE | 0.623 | 0.649 | 0.604 | 0.595 | 0.684 | 0.623 | 0.676 | 0.583 | 0.668 | 0.684 | 0.625 | 0.006 | 0.267 |

eTable 4: 95% confidence intervals for status based absolute and relative estimates

| Absolute estimates | | | | | | | | | | | | | | |
| --- | --- | --- | --- | --- | --- | --- | --- | --- | --- | --- | --- | --- | --- | --- |
| Baseline = No pain | | | | | | | | | | | | | | |
| Sex | Age | Est. | 1993 | | 1998 | | 2000 | | 2002 | | 2004 | | 2006 | |
|  |  |  | Lo | Hi | Lo | Hi | Lo | Hi | Lo | Hi | Lo | Hi | Lo | Hi |
| M | 70 | PFLE | 11.58 | 12.92 | 9.31 | 10.76 | 9.84 | 11.11 | 11.09 | 12.45 | 10.29 | 11.69 | 12.29 | 13.72 |
|  |  | NLPLE | 0.80 | 1.19 | 0.60 | 0.95 | 0.89 | 1.29 | 1.15 | 1.67 | 1.09 | 1.58 | 1.15 | 1.63 |
|  |  | LPLE | 0.89 | 1.34 | 1.14 | 1.86 | 1.21 | 1.79 | 1.51 | 2.19 | 1.93 | 2.70 | 1.46 | 2.07 |
|  |  | TLE | 13.55 | 15.09 | 11.47 | 13.01 | 12.33 | 13.76 | 14.19 | 15.91 | 13.83 | 15.47 | 15.36 | 16.91 |
|  | 80 | PFLE | 6.46 | 7.53 | 6.17 | 7.37 | 6.08 | 7.12 | 6.72 | 7.93 | 6.38 | 7.50 | 7.66 | 8.91 |
|  |  | NLPLE | 0.45 | 0.81 | 0.40 | 0.78 | 0.46 | 0.84 | 0.49 | 0.90 | 0.58 | 1.03 | 0.45 | 0.80 |
|  |  | LPLE | 0.50 | 0.91 | 0.60 | 1.20 | 0.66 | 1.15 | 0.80 | 1.45 | 1.00 | 1.70 | 0.57 | 1.08 |
|  |  | TLE | 7.72 | 8.89 | 7.49 | 8.85 | 7.50 | 8.64 | 8.44 | 9.85 | 8.34 | 9.72 | 9.03 | 10.49 |
|  | 90 | PFLE | 3.29 | 4.25 | 3.91 | 5.13 | 3.58 | 4.62 | 3.72 | 4.91 | 3.56 | 4.76 | 3.95 | 5.28 |
|  |  | NLPLE | 0.18 | 0.49 | 0.19 | 0.68 | 0.18 | 0.50 | 0.15 | 0.43 | 0.21 | 0.59 | 0.12 | 0.38 |
|  |  | LPLE | 0.21 | 0.56 | 0.22 | 0.76 | 0.27 | 0.70 | 0.31 | 0.86 | 0.39 | 0.96 | 0.16 | 0.51 |
|  |  | TLE | 3.86 | 5.00 | 4.55 | 6.14 | 4.21 | 5.49 | 4.40 | 5.85 | 4.51 | 5.99 | 4.42 | 6.02 |
| F | 70 | PFLE | 12.54 | 14.00 | 11.57 | 13.02 | 11.85 | 13.13 | 11.70 | 13.35 | 11.26 | 12.57 | 12.25 | 13.56 |
|  |  | NLPLE | 0.93 | 1.38 | 1.49 | 2.07 | 1.42 | 1.95 | 1.35 | 1.86 | 1.42 | 2.01 | 1.52 | 2.07 |
|  |  | LPLE | 2.08 | 2.84 | 2.62 | 3.41 | 2.55 | 3.32 | 2.88 | 3.74 | 3.29 | 4.23 | 3.07 | 3.93 |
|  |  | TLE | 16.01 | 17.89 | 16.39 | 17.87 | 16.30 | 17.85 | 16.58 | 18.55 | 16.65 | 18.20 | 17.50 | 18.98 |
|  | 80 | PFLE | 7.81 | 9.17 | 7.33 | 8.38 | 7.44 | 8.48 | 7.96 | 9.41 | 7.17 | 8.16 | 7.73 | 8.73 |
|  |  | NLPLE | 0.50 | 0.87 | 0.73 | 1.14 | 0.75 | 1.25 | 0.71 | 1.13 | 0.63 | 1.01 | 0.70 | 1.20 |
|  |  | LPLE | 1.32 | 1.99 | 1.48 | 2.06 | 1.34 | 1.93 | 1.40 | 2.12 | 1.70 | 2.44 | 1.59 | 2.28 |
|  |  | TLE | 9.95 | 11.66 | 9.94 | 11.21 | 9.93 | 11.18 | 10.53 | 12.20 | 9.89 | 11.25 | 10.44 | 11.80 |
|  | 90 | PFLE | 4.45 | 5.80 | 4.11 | 5.12 | 4.17 | 5.15 | 4.78 | 6.24 | 4.02 | 4.94 | 3.97 | 4.80 |
|  |  | NLPLE | 0.22 | 0.53 | 0.27 | 0.58 | 0.28 | 0.68 | 0.30 | 0.67 | 0.20 | 0.43 | 0.22 | 0.53 |
|  |  | LPLE | 0.67 | 1.28 | 0.62 | 1.13 | 0.53 | 1.02 | 0.52 | 1.10 | 0.61 | 1.19 | 0.57 | 1.05 |
|  |  | TLE | 5.65 | 7.27 | 5.25 | 6.56 | 5.22 | 6.47 | 5.85 | 7.64 | 5.04 | 6.29 | 5.00 | 6.23 |

Continued

Table e4: Continued

| Absolute estimates | | | | | | | | | | | | |
| --- | --- | --- | --- | --- | --- | --- | --- | --- | --- | --- | --- | --- |
| Baseline = No pain | | | | | | | | | | | | |
| Sex | Age | Est. | 2008 | | 2010 | | 2012 | | 2014 | | 2016 | |
|  |  |  | Lo | Hi | Lo | Hi | Lo | Hi | Lo | Hi | Lo | Hi |
| M | 70 | PFLE | 9.95 | 11.19 | 12.14 | 13.63 | 10.34 | 11.72 | 9.63 | 11.25 | 10.46 | 12.21 |
|  |  | NLPLE | 1.39 | 1.95 | 1.31 | 1.86 | 1.54 | 2.24 | 1.56 | 2.23 | 1.76 | 2.48 |
|  |  | LPLE | 1.71 | 2.48 | 1.86 | 2.62 | 1.92 | 2.63 | 1.62 | 2.36 | 2.12 | 3.10 |
|  |  | TLE | 13.66 | 14.97 | 15.92 | 17.47 | 14.41 | 15.78 | 13.44 | 15.02 | 15.08 | 16.87 |
|  | 80 | PFLE | 5.95 | 6.88 | 7.47 | 8.79 | 6.69 | 7.85 | 6.18 | 7.12 | 6.60 | 7.83 |
|  |  | NLPLE | 0.62 | 0.99 | 0.63 | 1.10 | 0.56 | 0.93 | 0.70 | 1.11 | 0.75 | 1.21 |
|  |  | LPLE | 0.80 | 1.33 | 0.85 | 1.58 | 0.77 | 1.24 | 0.61 | 1.01 | 0.77 | 1.29 |
|  |  | TLE | 7.77 | 8.79 | 9.43 | 10.91 | 8.41 | 9.56 | 7.86 | 8.87 | 8.49 | 9.79 |
|  | 90 | PFLE | 3.23 | 4.11 | 3.94 | 5.27 | 3.67 | 4.76 | 3.40 | 4.38 | 3.44 | 4.50 |
|  |  | NLPLE | 0.18 | 0.42 | 0.24 | 0.58 | 0.14 | 0.35 | 0.20 | 0.47 | 0.20 | 0.51 |
|  |  | LPLE | 0.24 | 0.62 | 0.27 | 0.84 | 0.20 | 0.49 | 0.14 | 0.38 | 0.15 | 0.46 |
|  |  | TLE | 3.89 | 4.85 | 4.74 | 6.30 | 4.14 | 5.32 | 3.93 | 4.98 | 3.93 | 5.33 |
| F | 70 | PFLE | 10.21 | 11.45 | 13.61 | 15.24 | 10.73 | 12.26 | 10.37 | 11.72 | 11.70 | 13.43 |
|  |  | NLPLE | 1.62 | 2.15 | 1.78 | 2.43 | 2.21 | 2.87 | 1.77 | 2.45 | 2.00 | 2.77 |
|  |  | LPLE | 3.35 | 4.15 | 3.07 | 3.98 | 4.23 | 5.43 | 3.53 | 4.46 | 3.41 | 4.44 |
|  |  | TLE | 15.74 | 17.07 | 19.15 | 21.02 | 17.99 | 19.64 | 16.37 | 17.82 | 17.85 | 19.70 |
|  | 80 | PFLE | 6.73 | 7.62 | 8.95 | 10.38 | 7.72 | 9.21 | 6.90 | 7.83 | 7.86 | 9.31 |
|  |  | NLPLE | 0.83 | 1.26 | 0.91 | 1.44 | 0.96 | 1.44 | 0.75 | 1.18 | 0.90 | 1.44 |
|  |  | LPLE | 1.42 | 2.06 | 1.59 | 2.42 | 2.07 | 3.08 | 1.63 | 2.30 | 1.43 | 2.14 |
|  |  | TLE | 9.32 | 10.45 | 11.97 | 13.67 | 11.30 | 12.87 | 9.73 | 10.84 | 3.41 | 4.44 |
|  | 90 | PFLE | 3.80 | 4.67 | 5.06 | 6.57 | 4.93 | 6.46 | 3.85 | 4.62 | 4.53 | 5.86 |
|  |  | NLPLE | 0.28 | 0.66 | 0.34 | 0.70 | 0.30 | 0.61 | 0.21 | 0.47 | 0.28 | 0.59 |
|  |  | LPLE | 0.42 | 0.86 | 0.59 | 1.24 | 0.78 | 1.57 | 0.52 | 0.99 | 0.40 | 0.83 |
|  |  | TLE | 4.75 | 5.80 | 6.24 | 8.10 | 6.30 | 8.05 | 4.79 | 5.79 | 5.47 | 6.96 |

Continued

Table e4: Continued

| Absolute estimates | | | | | | | | | | | | | | |
| --- | --- | --- | --- | --- | --- | --- | --- | --- | --- | --- | --- | --- | --- | --- |
| Baseline = Non-limiting pain | | | | | | | | | | | | | | |
| Sex | Age | Est. | 1993 | | 1998 | | 2000 | | 2002 | | 2004 | | 2006 | |
|  |  |  | Lo | Hi | Lo | Hi | Lo | Hi | Lo | Hi | Lo | Hi | Lo | Hi |
| M | 70 | PFLE | 8.50 | 10.32 | 6.20 | 8.39 | 6.16 | 8.08 | 7.70 | 9.63 | 6.86 | 8.50 | 8.45 | 10.40 |
|  |  | NLPLE | 2.92 | 3.71 | 2.72 | 3.64 | 3.27 | 4.28 | 3.54 | 4.58 | 3.38 | 4.21 | 3.48 | 4.24 |
|  |  | LPLE | 1.14 | 1.73 | 1.41 | 2.41 | 1.78 | 2.78 | 1.71 | 2.70 | 2.37 | 3.37 | 2.00 | 2.87 |
|  |  | TLE | 13.26 | 14.97 | 11.17 | 13.31 | 12.35 | 13.93 | 13.80 | 15.86 | 13.36 | 15.10 | 14.58 | 16.48 |
|  | 80 | PFLE | 3.42 | 4.68 | 3.26 | 4.59 | 2.84 | 4.17 | 3.60 | 4.92 | 2.96 | 4.33 | 4.65 | 6.03 |
|  |  | NLPLE | 2.62 | 3.28 | 2.62 | 3.32 | 2.72 | 3.37 | 2.75 | 3.49 | 2.90 | 3.70 | 2.73 | 3.37 |
|  |  | LPLE | 0.84 | 1.39 | 0.74 | 1.50 | 1.00 | 1.84 | 1.08 | 1.92 | 1.41 | 2.30 | 0.83 | 1.49 |
|  |  | TLE | 7.41 | 8.83 | 7.11 | 8.70 | 7.26 | 8.66 | 8.11 | 9.64 | 7.99 | 9.52 | 8.81 | 10.20 |
|  | 90 | PFLE | 0.78 | 1.80 | 0.89 | 2.51 | 0.85 | 2.28 | 1.06 | 2.42 | 0.82 | 1.99 | 1.71 | 3.22 |
|  |  | NLPLE | 1.97 | 2.75 | 2.01 | 3.05 | 1.95 | 2.79 | 1.92 | 2.75 | 2.16 | 3.28 | 2.03 | 2.84 |
|  |  | LPLE | 0.39 | 1.05 | 0.23 | 0.95 | 0.30 | 1.10 | 0.40 | 1.53 | 0.60 | 1.61 | 0.21 | 0.80 |
|  |  | TLE | 3.58 | 5.00 | 3.54 | 5.99 | 3.57 | 5.35 | 3.95 | 5.86 | 4.08 | 6.09 | 4.38 | 6.19 |
| F | 70 | PFLE | 9.38 | 10.91 | 7.08 | 8.98 | 8.38 | 9.90 | 7.74 | 9.66 | 7.95 | 9.50 | 8.75 | 10.40 |
|  |  | NLPLE | 3.08 | 3.71 | 4.23 | 5.43 | 3.56 | 4.26 | 3.65 | 4.49 | 4.13 | 5.17 | 3.94 | 4.76 |
|  |  | LPLE | 2.75 | 3.61 | 3.11 | 4.46 | 3.13 | 4.25 | 3.30 | 4.47 | 3.73 | 4.75 | 3.39 | 4.45 |
|  |  | TLE | 15.93 | 17.69 | 15.63 | 17.65 | 15.96 | 17.55 | 15.61 | 17.81 | 16.72 | 18.44 | 16.94 | 18.76 |
|  | 80 | PFLE | 6.31 | 2.63 | 3.77 | 5.01 | 4.48 | 5.63 | 4.58 | 6.18 | 4.09 | 5.21 | 4.62 | 5.78 |
|  |  | NLPLE | 3.24 | 1.95 | 3.12 | 3.85 | 2.99 | 3.79 | 2.87 | 3.47 | 3.02 | 3.63 | 3.07 | 3.88 |
|  |  | LPLE | 2.76 | 9.47 | 1.88 | 2.72 | 1.66 | 2.41 | 1.70 | 2.54 | 2.36 | 3.34 | 2.00 | 2.85 |
|  |  | TLE | 11.79 | 4.96 | 9.47 | 10.94 | 9.73 | 11.18 | 9.69 | 11.59 | 10.08 | 11.58 | 10.32 | 11.78 |
|  | 90 | PFLE | 2.09 | 3.49 | 1.40 | 2.59 | 1.77 | 3.00 | 2.15 | 3.69 | 1.55 | 2.55 | 1.71 | 2.81 |
|  |  | NLPLE | 2.16 | 2.91 | 2.10 | 2.83 | 2.40 | 3.44 | 2.12 | 2.81 | 2.04 | 2.70 | 2.28 | 3.23 |
|  |  | LPLE | 1.12 | 2.05 | 0.80 | 1.65 | 0.63 | 1.33 | 0.61 | 1.35 | 1.21 | 2.36 | 0.97 | 1.93 |
|  |  | TLE | 5.86 | 7.77 | 4.76 | 6.50 | 5.30 | 7.03 | 5.30 | 7.20 | 5.33 | 6.92 | 5.44 | 7.17 |

Continued

Table e4: Continued

| Absolute estimates | | | | | | | | | | | | |
| --- | --- | --- | --- | --- | --- | --- | --- | --- | --- | --- | --- | --- |
| Baseline = Non-limiting pain | | | | | | | | | | | | |
| Sex | Age | Est. | 2008 | | 2010 | | 2012 | | 2014 | | 2016 | |
|  |  |  | Lo | Hi | Lo | Hi | Lo | Hi | Lo | Hi | Lo | Hi |
| M | 70 | PFLE | 6.49 | 8.16 | 7.90 | 9.72 | 5.79 | 7.57 | 5.92 | 7.52 | 6.59 | 8.62 |
|  |  | NLPLE | 4.05 | 5.06 | 3.78 | 4.62 | 4.53 | 5.88 | 4.08 | 5.12 | 4.75 | 6.23 |
|  |  | LPLE | 1.92 | 2.84 | 2.36 | 3.42 | 2.24 | 3.19 | 2.26 | 3.30 | 2.42 | 3.72 |
|  |  | TLE | 13.39 | 15.01 | 14.67 | 16.90 | 13.48 | 15.54 | 13.14 | 14.87 | 15.15 | 16.91 |
|  | 80 | PFLE | 2.54 | 3.61 | 4.17 | 5.52 | 2.99 | 4.28 | 2.76 | 3.81 | 3.21 | 4.50 |
|  |  | NLPLE | 3.11 | 3.90 | 2.97 | 3.69 | 3.17 | 4.03 | 3.19 | 4.02 | 3.39 | 4.24 |
|  |  | LPLE | 1.08 | 1.79 | 1.29 | 2.14 | 1.14 | 1.82 | 1.00 | 1.72 | 1.17 | 1.97 |
|  |  | TLE | 7.37 | 8.70 | 9.14 | 10.61 | 8.01 | 9.32 | 7.50 | 8.80 | 8.48 | 9.92 |
|  | 90 | PFLE | 0.50 | 1.35 | 1.65 | 2.93 | 1.09 | 2.25 | 0.69 | 1.61 | 0.95 | 2.01 |
|  |  | NLPLE | 2.02 | 2.94 | 2.22 | 3.17 | 2.15 | 3.09 | 2.16 | 2.95 | 2.07 | 2.99 |
|  |  | LPLE | 0.39 | 1.09 | 0.50 | 1.38 | 0.40 | 1.04 | 0.26 | 0.87 | 0.38 | 0.99 |
|  |  | TLE | 3.18 | 4.83 | 4.89 | 6.73 | 4.16 | 5.64 | 3.55 | 4.92 | 3.79 | 5.33 |
| F | 70 | PFLE | 6.35 | 7.69 | 9.96 | 11.82 | 7.00 | 8.63 | 6.13 | 7.67 | 8.31 | 10.06 |
|  |  | NLPLE | 3.97 | 4.87 | 4.35 | 5.23 | 4.64 | 5.60 | 4.44 | 5.38 | 4.60 | 5.71 |
|  |  | LPLE | 4.16 | 5.28 | 3.52 | 4.56 | 5.20 | 6.57 | 4.54 | 5.73 | 3.75 | 4.97 |
|  |  | TLE | 15.21 | 16.86 | 18.85 | 20.80 | 17.91 | 19.69 | 16.07 | 17.63 | 17.74 | 19.57 |
|  | 80 | PFLE | 3.52 | 4.58 | 5.36 | 6.81 | 4.03 | 5.55 | 3.68 | 4.63 | 4.25 | 5.86 |
|  |  | NLPLE | 3.23 | 3.94 | 3.38 | 4.18 | 3.49 | 4.15 | 3.40 | 4.07 | 3.34 | 4.20 |
|  |  | LPLE | 1.70 | 2.51 | 2.22 | 3.21 | 2.73 | 3.86 | 2.18 | 3.06 | 1.95 | 2.76 |
|  |  | TLE | 9.06 | 10.30 | 11.68 | 13.51 | 11.00 | 12.70 | 9.82 | 11.10 | 10.42 | 12.10 |
|  | 90 | PFLE | 1.35 | 2.17 | 2.05 | 3.25 | 1.49 | 2.91 | 1.48 | 2.31 | 1.41 | 2.92 |
|  |  | NLPLE | 2.25 | 2.97 | 2.49 | 3.33 | 2.32 | 3.13 | 2.30 | 3.04 | 2.26 | 3.16 |
|  |  | LPLE | 0.42 | 1.00 | 1.12 | 2.23 | 0.97 | 2.04 | 0.73 | 1.44 | 0.88 | 1.84 |
|  |  | TLE | 4.38 | 5.76 | 6.12 | 8.05 | 5.34 | 7.29 | 4.90 | 6.28 | 5.03 | 7.01 |

Continued

Table e4: Continued

| Absolute estimates | | | | | | | | | | | | | | |
| --- | --- | --- | --- | --- | --- | --- | --- | --- | --- | --- | --- | --- | --- | --- |
| Baseline = Limiting pain | | | | | | | | | | | | | | |
| Sex | Age | Est. | 1993 | | 1998 | | 2000 | | 2002 | | 2004 | | 2006 | |
|  |  |  | Lo | Hi | Lo | Hi | Lo | Hi | Lo | Hi | Lo | Hi | Lo | Hi |
| M | 70 | PFLE | 6.62 | 8.46 | 3.75 | 6.62 | 5.36 | 7.42 | 6.00 | 8.16 | 4.54 | 6.41 | 6.81 | 8.83 |
|  |  | NLPLE | 1.07 | 1.72 | 0.44 | 0.95 | 0.89 | 1.52 | 1.00 | 1.64 | 0.94 | 1.51 | 1.18 | 1.85 |
|  |  | LPLE | 3.44 | 4.33 | 4.39 | 6.32 | 4.30 | 5.64 | 4.78 | 6.10 | 5.46 | 7.01 | 4.70 | 5.88 |
|  |  | TLE | 11.89 | 13.75 | 9.56 | 12.72 | 11.37 | 13.47 | 12.77 | 14.87 | 11.81 | 14.04 | 13.48 | 15.61 |
|  | 80 | PFLE | 2.58 | 3.71 | 2.03 | 3.30 | 1.59 | 2.60 | 2.61 | 3.83 | 2.07 | 3.08 | 3.51 | 4.77 |
|  |  | NLPLE | 0.49 | 0.92 | 0.31 | 0.79 | 0.44 | 0.92 | 0.46 | 0.98 | 0.49 | 1.07 | 0.49 | 0.96 |
|  |  | LPLE | 2.94 | 3.56 | 3.53 | 4.77 | 3.63 | 4.65 | 3.87 | 4.94 | 4.18 | 5.24 | 3.34 | 4.14 |
|  |  | TLE | 6.42 | 7.75 | 6.49 | 8.38 | 6.22 | 7.59 | 7.43 | 9.10 | 7.19 | 8.79 | 7.82 | 9.33 |
|  | 90 | PFLE | 0.70 | 1.57 | 0.60 | 1.94 | 0.24 | 0.86 | 0.72 | 1.80 | 0.67 | 1.67 | 1.28 | 2.40 |
|  |  | NLPLE | 0.13 | 0.48 | 0.14 | 0.99 | 0.12 | 0.68 | 0.14 | 0.67 | 0.17 | 0.81 | 0.14 | 0.53 |
|  |  | LPLE | 2.09 | 2.93 | 2.53 | 4.20 | 2.28 | 3.37 | 2.66 | 4.19 | 2.84 | 4.25 | 2.25 | 3.02 |
|  |  | TLE | 3.19 | 4.54 | 3.61 | 6.25 | 2.87 | 4.49 | 3.94 | 5.88 | 3.96 | 5.86 | 4.03 | 5.50 |
| F | 70 | PFLE | 7.79 | 9.56 | 6.08 | 8.03 | 6.63 | 8.23 | 6.71 | 8.53 | 6.19 | 7.81 | 6.44 | 8.04 |
|  |  | NLPLE | 1.01 | 1.47 | 1.56 | 2.32 | 1.36 | 1.91 | 1.33 | 1.89 | 1.47 | 2.12 | 1.45 | 2.09 |
|  |  | LPLE | 5.58 | 6.70 | 6.14 | 7.46 | 6.41 | 7.71 | 6.58 | 7.84 | 7.35 | 8.81 | 6.58 | 7.83 |
|  |  | TLE | 15.00 | 17.05 | 14.37 | 16.78 | 14.99 | 16.97 | 15.31 | 17.46 | 15.92 | 17.85 | 15.22 | 17.27 |
|  | 80 | PFLE | 3.63 | 4.89 | 3.12 | 4.17 | 3.24 | 4.29 | 3.61 | 5.02 | 2.97 | 4.04 | 3.42 | 4.46 |
|  |  | NLPLE | 0.62 | 1.03 | 0.79 | 1.28 | 0.81 | 1.35 | 0.71 | 1.16 | 0.62 | 1.02 | 0.76 | 1.26 |
|  |  | LPLE | 4.43 | 5.34 | 4.65 | 5.55 | 4.49 | 5.34 | 4.71 | 5.62 | 5.19 | 6.21 | 4.79 | 5.69 |
|  |  | TLE | 9.14 | 10.74 | 9.00 | 10.48 | 8.98 | 10.49 | 9.53 | 11.28 | 9.27 | 10.77 | 9.51 | 10.96 |
|  | 90 | PFLE | 1.24 | 2.27 | 1.11 | 2.02 | 1.07 | 1.98 | 1.38 | 2.67 | 0.98 | 1.82 | 1.33 | 2.22 |
|  |  | NLPLE | 0.31 | 0.73 | 0.28 | 0.70 | 0.35 | 0.93 | 0.28 | 0.72 | 0.18 | 0.48 | 0.31 | 0.79 |
|  |  | LPLE | 3.13 | 4.22 | 3.26 | 4.30 | 2.83 | 3.82 | 3.18 | 4.14 | 3.17 | 4.23 | 3.27 | 4.38 |
|  |  | TLE | 5.11 | 6.84 | 5.05 | 6.62 | 4.54 | 6.39 | 5.16 | 6.97 | 4.68 | 6.17 | 5.36 | 6.87 |

Continued

Table e4: Continued

| Absolute estimates | | | | | | | | | | | | |
| --- | --- | --- | --- | --- | --- | --- | --- | --- | --- | --- | --- | --- |
| Baseline = Limiting pain | | | | | | | | | | | | |
| Sex | Age | Est. | 2008 | | 2010 | | 2012 | | 2014 | | 2016 | |
|  |  |  | Lo | Hi | Lo | Hi | Lo | Hi | Lo | Hi | Lo | Hi |
| M | 70 | PFLE | 4.81 | 6.42 | 6.72 | 8.59 | 5.23 | 6.95 | 4.52 | 6.34 | 5.64 | 7.67 |
|  |  | NLPLE | 1.63 | 2.38 | 1.45 | 2.16 | 1.61 | 2.55 | 1.54 | 2.34 | 1.95 | 3.08 |
|  |  | LPLE | 5.06 | 6.40 | 5.23 | 6.40 | 5.33 | 6.67 | 4.69 | 5.93 | 5.52 | 7.14 |
|  |  | TLE | 12.35 | 14.25 | 14.18 | 16.30 | 13.09 | 15.08 | 11.47 | 13.78 | 14.51 | 16.64 |
|  | 80 | PFLE | 1.73 | 2.71 | 3.12 | 4.45 | 2.02 | 3.30 | 1.76 | 2.64 | 2.30 | 3.46 |
|  |  | NLPLE | 0.63 | 1.12 | 0.65 | 1.15 | 0.65 | 1.23 | 0.70 | 1.16 | 0.93 | 1.54 |
|  |  | LPLE | 3.86 | 4.89 | 4.06 | 5.11 | 3.97 | 4.86 | 3.51 | 4.25 | 4.02 | 5.03 |
|  |  | TLE | 6.69 | 8.08 | 8.45 | 9.89 | 7.28 | 8.63 | 6.42 | 7.62 | 7.80 | 9.28 |
|  | 90 | PFLE | 0.35 | 1.08 | 0.90 | 2.08 | 0.48 | 1.24 | 0.42 | 1.00 | 0.49 | 1.26 |
|  |  | NLPLE | 0.14 | 0.46 | 0.20 | 0.62 | 0.16 | 0.61 | 0.21 | 0.57 | 0.24 | 0.76 |
|  |  | LPLE | 2.53 | 3.54 | 2.82 | 4.22 | 2.44 | 3.49 | 2.30 | 3.17 | 2.41 | 3.33 |
|  |  | TLE | 3.25 | 4.76 | 4.36 | 6.27 | 3.42 | 4.82 | 3.15 | 4.29 | 3.44 | 4.88 |
| F | 70 | PFLE | 5.43 | 6.82 | 8.49 | 10.20 | 5.75 | 7.30 | 4.83 | 6.28 | 6.42 | 8.30 |
|  |  | NLPLE | 1.56 | 2.16 | 1.80 | 2.46 | 1.90 | 2.65 | 1.61 | 2.29 | 1.92 | 2.78 |
|  |  | LPLE | 6.97 | 8.06 | 6.84 | 7.99 | 8.36 | 9.95 | 7.49 | 8.88 | 7.61 | 9.19 |
|  |  | TLE | 14.63 | 16.23 | 17.89 | 19.87 | 16.82 | 19.04 | 14.47 | 16.79 | 16.87 | 19.12 |
|  | 80 | PFLE | 2.68 | 3.58 | 4.28 | 5.74 | 2.96 | 4.39 | 2.55 | 3.38 | 3.30 | 4.57 |
|  |  | NLPLE | 0.81 | 1.28 | 1.02 | 1.61 | 0.92 | 1.41 | 0.77 | 1.28 | 0.91 | 1.51 |
|  |  | LPLE | 4.76 | 5.64 | 5.07 | 6.10 | 5.79 | 7.24 | 5.17 | 6.01 | 5.07 | 6.07 |
|  |  | TLE | 8.73 | 9.95 | 11.05 | 12.72 | 10.44 | 12.25 | 8.92 | 10.27 | 9.86 | 11.50 |
|  | 90 | PFLE | 0.95 | 1.60 | 1.54 | 2.84 | 1.16 | 2.37 | 0.92 | 1.60 | 1.11 | 2.16 |
|  |  | NLPLE | 0.28 | 0.71 | 0.48 | 1.05 | 0.30 | 0.73 | 0.28 | 0.70 | 0.30 | 0.76 |
|  |  | LPLE | 3.06 | 4.15 | 3.47 | 4.75 | 3.77 | 5.29 | 3.27 | 4.31 | 3.06 | 3.96 |
|  |  | TLE | 4.58 | 6.07 | 6.08 | 7.84 | 5.69 | 7.78 | 4.83 | 6.30 | 4.89 | 6.42 |

Continued

Table e4: Continued

| Relative estimates | | | | | | | | | | | | | | |
| --- | --- | --- | --- | --- | --- | --- | --- | --- | --- | --- | --- | --- | --- | --- |
| Baseline = No pain | | | | | | | | | | | | | | |
| Sex | Age | Est. | 1993 | | 1998 | | 2000 | | 2002 | | 2004 | | 2006 | |
|  |  |  | Lo | Hi | Lo | Hi | Lo | Hi | Lo | Hi | Lo | Hi | Lo | Hi |
| M | 70 | PFLE | 80.9% | 90.3% | 76.0% | 87.8% | 75.5% | 85.2% | 73.8% | 82.8% | 70.4% | 79.9% | 76.5% | 85.4% |
|  |  | NLPLE | 5.6% | 8.3% | 4.9% | 7.7% | 6.8% | 9.9% | 7.6% | 11.1% | 7.4% | 10.8% | 7.1% | 10.1% |
|  |  | LPLE | 6.2% | 9.3% | 9.3% | 15.2% | 9.3% | 13.8% | 11.6% | 16.8% | 13.2% | 18.5% | 9.1% | 12.9% |
|  | 80 | PFLE | 78.2% | 91.2% | 75.8% | 90.6% | 75.3% | 88.2% | 73.8% | 87.2% | 71.1% | 83.5% | 79.6% | 92.6% |
|  |  | NLPLE | 5.5% | 9.8% | 5.0% | 9.6% | 5.7% | 10.4% | 5.3% | 9.9% | 6.5% | 11.5% | 4.7% | 8.3% |
|  |  | LPLE | 6.1% | 11.1% | 7.4% | 14.7% | 8.2% | 14.3% | 8.7% | 16.0% | 11.1% | 18.9% | 5.9% | 11.2% |
|  | 90 | PFLE | 75.7% | 97.8% | 74.6% | 97.7% | 74.0% | 95.4% | 73.2% | 96.6% | 69.7% | 93.2% | 77.7% | 103.9% |
|  |  | NLPLE | 4.1% | 11.2% | 3.6% | 12.9% | 3.6% | 10.2% | 3.0% | 8.5% | 4.2% | 11.5% | 2.3% | 7.4% |
|  |  | LPLE | 4.8% | 12.9% | 4.2% | 14.5% | 5.5% | 14.4% | 6.1% | 17.0% | 7.5% | 18.7% | 3.1% | 9.9% |
| F | 70 | PFLE | 74.7% | 83.4% | 68.0% | 76.6% | 69.4% | 76.9% | 67.2% | 76.7% | 65.0% | 72.6% | 67.3% | 74.5% |
|  |  | NLPLE | 5.6% | 8.2% | 8.7% | 12.2% | 8.3% | 11.4% | 7.7% | 10.7% | 8.2% | 11.6% | 8.3% | 11.4% |
|  |  | LPLE | 12.4% | 16.9% | 15.4% | 20.1% | 14.9% | 19.5% | 16.6% | 21.5% | 19.0% | 24.4% | 16.9% | 21.6% |
|  | 80 | PFLE | 72.7% | 85.4% | 69.6% | 79.5% | 70.8% | 80.8% | 70.6% | 83.4% | 68.4% | 77.9% | 70.2% | 79.3% |
|  |  | NLPLE | 4.7% | 8.1% | 6.9% | 10.9% | 7.1% | 11.9% | 6.3% | 10.1% | 6.0% | 9.6% | 6.3% | 10.9% |
|  |  | LPLE | 12.3% | 18.5% | 14.0% | 19.6% | 12.7% | 18.4% | 12.4% | 18.8% | 16.2% | 23.2% | 14.4% | 20.7% |
|  | 90 | PFLE | 69.9% | 91.1% | 70.7% | 87.9% | 72.3% | 89.3% | 71.6% | 93.5% | 71.6% | 88.1% | 72.5% | 87.5% |
|  |  | NLPLE | 3.5% | 8.3% | 4.6% | 10.0% | 4.9% | 11.7% | 4.4% | 10.1% | 3.6% | 7.7% | 4.0% | 9.7% |
|  |  | LPLE | 10.5% | 20.1% | 10.6% | 19.4% | 9.1% | 17.7% | 7.9% | 16.4% | 10.9% | 21.2% | 10.5% | 19.1% |

Continued

Table e4: Continued

| Relative estimates | | | | | | | | | | | | |
| --- | --- | --- | --- | --- | --- | --- | --- | --- | --- | --- | --- | --- |
| Baseline = No pain | | | | | | | | | | | | |
| Sex | Age | Est. | 2008 | | 2010 | | 2012 | | 2014 | | 2016 | |
|  |  |  | Lo | Hi | Lo | Hi | Lo | Hi | Lo | Hi | Lo | Hi |
| M | 70 | PFLE | 69.5% | 78.1% | 73.0% | 81.9% | 68.5% | 77.6% | 67.8% | 79.2% | 65.6% | 76.6% |
|  |  | NLPLE | 9.7% | 13.6% | 7.9% | 11.2% | 10.2% | 14.8% | 11.0% | 15.7% | 11.0% | 15.6% |
|  |  | LPLE | 11.9% | 17.3% | 11.2% | 15.7% | 12.7% | 17.4% | 11.4% | 16.6% | 13.3% | 19.4% |
|  | 80 | PFLE | 72.4% | 83.6% | 73.8% | 86.9% | 75.0% | 87.9% | 74.4% | 85.7% | 72.8% | 86.4% |
|  |  | NLPLE | 7.5% | 12.0% | 6.2% | 10.9% | 6.3% | 10.4% | 8.5% | 13.4% | 8.2% | 13.4% |
|  |  | LPLE | 9.7% | 16.1% | 8.4% | 15.6% | 8.6% | 13.8% | 7.3% | 12.2% | 8.5% | 14.2% |
|  | 90 | PFLE | 75.7% | 96.4% | 72.6% | 97.2% | 78.1% | 101.3% | 77.6% | 100.0% | 76.9% | 100.5% |
|  |  | NLPLE | 4.2% | 9.9% | 4.4% | 10.7% | 3.0% | 7.4% | 4.6% | 10.7% | 4.5% | 11.5% |
|  |  | LPLE | 5.6% | 14.4% | 5.0% | 15.4% | 4.2% | 10.4% | 3.2% | 8.7% | 3.4% | 10.4% |
| F | 70 | PFLE | 62.3% | 69.8% | 68.2% | 76.4% | 57.1% | 65.3% | 60.6% | 68.5% | 62.7% | 72.0% |
|  |  | NLPLE | 9.9% | 13.1% | 8.9% | 12.2% | 11.8% | 15.3% | 10.4% | 14.3% | 10.7% | 14.8% |
|  |  | LPLE | 20.5% | 25.3% | 15.4% | 19.9% | 22.5% | 28.9% | 20.6% | 26.1% | 18.3% | 23.8% |
|  | 80 | PFLE | 68.0% | 77.0% | 70.5% | 81.8% | 63.9% | 76.2% | 67.0% | 76.0% | 68.5% | 81.2% |
|  |  | NLPLE | 8.4% | 12.8% | 7.1% | 11.4% | 8.0% | 11.9% | 7.3% | 11.5% | 7.8% | 12.6% |
|  |  | LPLE | 14.3% | 20.8% | 12.5% | 19.1% | 17.2% | 25.5% | 15.8% | 22.3% | 12.4% | 18.6% |
|  | 90 | PFLE | 72.7% | 89.3% | 71.5% | 92.8% | 69.1% | 90.6% | 73.1% | 87.7% | 74.3% | 96.1% |
|  |  | NLPLE | 5.3% | 12.6% | 4.8% | 10.0% | 4.2% | 8.6% | 3.9% | 9.0% | 4.6% | 9.7% |
|  |  | LPLE | 8.1% | 16.4% | 8.4% | 17.5% | 10.9% | 22.0% | 9.9% | 18.8% | 6.5% | 13.5% |

Continued

Table e4: Continued

| Relative estimates | | | | | | | | | | | | | | |
| --- | --- | --- | --- | --- | --- | --- | --- | --- | --- | --- | --- | --- | --- | --- |
| Baseline = Non-limiting pain | | | | | | | | | | | | | | |
| Sex | Age | Est. | 1993 | | 1998 | | 2000 | | 2002 | | 2004 | | 2006 | |
|  |  |  | Lo | Hi | Lo | Hi | Lo | Hi | Lo | Hi | Lo | Hi | Lo | Hi |
| M | 70 | PFLE | 60.4% | 73.2% | 50.2% | 67.8% | 46.8% | 61.4% | 52.3% | 65.4% | 48.1% | 59.5% | 54.4% | 66.9% |
|  |  | NLPLE | 20.8% | 26.3% | 22.0% | 29.4% | 24.8% | 32.5% | 24.0% | 31.1% | 23.6% | 29.5% | 22.4% | 27.3% |
|  |  | LPLE | 8.1% | 12.3% | 11.4% | 19.5% | 13.5% | 21.1% | 11.6% | 18.3% | 16.6% | 23.6% | 12.9% | 18.5% |
|  | 80 | PFLE | 42.7% | 58.6% | 41.2% | 58.1% | 35.8% | 52.6% | 40.8% | 55.7% | 34.0% | 49.8% | 49.5% | 64.2% |
|  |  | NLPLE | 32.8% | 41.0% | 33.1% | 41.9% | 34.4% | 42.5% | 31.1% | 39.5% | 33.3% | 42.5% | 29.1% | 35.9% |
|  |  | LPLE | 10.5% | 17.4% | 9.4% | 19.0% | 12.6% | 23.3% | 12.2% | 21.7% | 16.3% | 26.4% | 8.8% | 15.8% |
|  | 90 | PFLE | 18.5% | 42.8% | 19.4% | 54.5% | 19.3% | 51.8% | 21.7% | 49.6% | 16.8% | 40.8% | 33.0% | 62.4% |
|  |  | NLPLE | 46.7% | 65.3% | 43.5% | 66.1% | 44.3% | 63.5% | 39.4% | 56.4% | 44.2% | 67.1% | 39.4% | 54.9% |
|  |  | LPLE | 9.3% | 25.0% | 4.9% | 20.6% | 6.9% | 25.0% | 8.3% | 31.3% | 12.4% | 32.9% | 4.2% | 15.4% |
| F | 70 | PFLE | 56.2% | 65.3% | 42.8% | 54.3% | 50.0% | 59.1% | 46.6% | 58.1% | 45.4% | 54.3% | 49.4% | 58.7% |
|  |  | NLPLE | 18.5% | 22.2% | 25.6% | 32.8% | 21.3% | 25.5% | 22.0% | 27.0% | 23.6% | 29.6% | 22.3% | 26.9% |
|  |  | LPLE | 16.4% | 21.6% | 18.8% | 27.0% | 18.7% | 25.4% | 19.9% | 26.9% | 21.3% | 27.1% | 19.2% | 25.1% |
|  | 80 | PFLE | 58.2% | 24.2% | 37.2% | 49.3% | 43.1% | 54.1% | 43.2% | 58.3% | 38.1% | 48.5% | 42.2% | 52.8% |
|  |  | NLPLE | 29.9% | 18.0% | 30.7% | 37.9% | 28.8% | 36.4% | 27.1% | 32.7% | 28.1% | 33.8% | 28.1% | 35.4% |
|  |  | LPLE | 25.5% | 87.4% | 18.5% | 26.8% | 16.0% | 23.2% | 16.1% | 23.9% | 21.9% | 31.1% | 18.2% | 26.0% |
|  | 90 | PFLE | 30.8% | 51.4% | 25.0% | 46.1% | 29.2% | 49.7% | 34.1% | 58.6% | 25.6% | 42.1% | 27.5% | 45.2% |
|  |  | NLPLE | 31.8% | 42.9% | 37.5% | 50.5% | 39.8% | 56.9% | 33.6% | 44.7% | 33.8% | 44.7% | 36.7% | 51.9% |
|  |  | LPLE | 16.6% | 30.3% | 14.3% | 29.4% | 10.5% | 22.0% | 9.7% | 21.4% | 20.1% | 39.0% | 15.6% | 31.1% |

Continued

Table e4: Continued

| Relative estimates | | | | | | | | | | | | |
| --- | --- | --- | --- | --- | --- | --- | --- | --- | --- | --- | --- | --- |
| Baseline = Non-limiting pain | | | | | | | | | | | | |
| Sex | Age | Est. | 2008 | | 2010 | | 2012 | | 2014 | | 2016 | |
|  |  |  | Lo | Hi | Lo | Hi | Lo | Hi | Lo | Hi | Lo | Hi |
| M | 70 | PFLE | 46.0% | 57.8% | 50.0% | 61.5% | 39.9% | 52.2% | 42.3% | 53.8% | 41.3% | 54.0% |
|  |  | NLPLE | 28.7% | 35.8% | 23.9% | 29.2% | 31.2% | 40.6% | 29.2% | 36.6% | 29.8% | 39.0% |
|  |  | LPLE | 13.6% | 20.1% | 14.9% | 21.6% | 15.4% | 22.0% | 16.2% | 23.6% | 15.2% | 23.3% |
|  | 80 | PFLE | 32.2% | 45.7% | 42.6% | 56.4% | 34.6% | 49.6% | 34.0% | 47.1% | 35.3% | 49.5% |
|  |  | NLPLE | 39.3% | 49.3% | 30.3% | 37.7% | 36.7% | 46.6% | 39.4% | 49.7% | 37.3% | 46.6% |
|  |  | LPLE | 13.7% | 22.6% | 13.2% | 21.9% | 13.2% | 21.1% | 12.4% | 21.3% | 12.9% | 21.7% |
|  | 90 | PFLE | 12.7% | 34.1% | 28.8% | 51.1% | 22.6% | 46.6% | 17.0% | 39.7% | 21.0% | 44.5% |
|  |  | NLPLE | 51.1% | 74.4% | 38.6% | 55.2% | 44.5% | 63.9% | 53.1% | 72.7% | 45.8% | 66.3% |
|  |  | LPLE | 9.9% | 27.5% | 8.7% | 24.0% | 8.3% | 21.5% | 6.3% | 21.4% | 8.4% | 22.0% |
| F | 70 | PFLE | 39.5% | 47.8% | 50.5% | 59.9% | 37.3% | 46.0% | 36.3% | 45.4% | 44.8% | 54.3% |
|  |  | NLPLE | 24.7% | 30.2% | 22.0% | 26.5% | 24.7% | 29.8% | 26.3% | 31.9% | 24.8% | 30.8% |
|  |  | LPLE | 25.8% | 32.8% | 17.9% | 23.1% | 27.7% | 35.0% | 26.9% | 33.9% | 20.2% | 26.8% |
|  | 80 | PFLE | 36.4% | 47.4% | 43.1% | 54.7% | 33.9% | 46.7% | 35.2% | 44.4% | 37.9% | 52.2% |
|  |  | NLPLE | 33.4% | 40.8% | 27.1% | 33.6% | 29.3% | 34.9% | 32.6% | 39.0% | 29.7% | 37.3% |
|  |  | LPLE | 17.6% | 26.0% | 17.9% | 25.8% | 23.0% | 32.5% | 20.9% | 29.3% | 17.4% | 24.6% |
|  | 90 | PFLE | 27.2% | 43.6% | 29.2% | 46.3% | 23.8% | 46.4% | 26.6% | 41.4% | 23.4% | 48.6% |
|  |  | NLPLE | 45.2% | 59.9% | 35.4% | 47.4% | 36.9% | 49.8% | 41.4% | 54.7% | 37.6% | 52.6% |
|  |  | LPLE | 8.5% | 20.2% | 15.9% | 31.8% | 15.4% | 32.4% | 13.2% | 25.9% | 14.7% | 30.7% |

Continued

Table e4: Continued

| Relative estimates | | | | | | | | | | | | | | |
| --- | --- | --- | --- | --- | --- | --- | --- | --- | --- | --- | --- | --- | --- | --- |
| Baseline = Limiting pain | | | | | | | | | | | | | | |
| Sex | Age | Est. | 1993 | | 1998 | | 2000 | | 2002 | | 2004 | | 2006 | |
|  |  |  | Lo | Hi | Lo | Hi | Lo | Hi | Lo | Hi | Lo | Hi | Lo | Hi |
| M | 70 | PFLE | 51.7% | 66.1% | 33.7% | 59.4% | 42.9% | 59.5% | 43.3% | 58.9% | 35.2% | 49.8% | 46.7% | 60.6% |
|  |  | NLPLE | 8.4% | 13.5% | 3.9% | 8.5% | 7.1% | 12.2% | 7.2% | 11.8% | 7.3% | 11.7% | 8.1% | 12.7% |
|  |  | LPLE | 26.9% | 33.9% | 39.4% | 56.8% | 34.4% | 45.2% | 34.5% | 44.1% | 42.4% | 54.4% | 32.2% | 40.4% |
|  | 80 | PFLE | 37.0% | 53.1% | 28.1% | 45.8% | 23.2% | 37.9% | 31.6% | 46.3% | 26.1% | 38.8% | 41.2% | 55.9% |
|  |  | NLPLE | 7.1% | 13.1% | 4.4% | 11.0% | 6.4% | 13.4% | 5.6% | 11.8% | 6.1% | 13.5% | 5.8% | 11.3% |
|  |  | LPLE | 42.0% | 50.9% | 49.0% | 66.2% | 53.0% | 67.8% | 46.8% | 59.7% | 52.7% | 66.1% | 39.2% | 48.6% |
|  | 90 | PFLE | 18.7% | 41.9% | 13.1% | 42.1% | 6.8% | 24.3% | 14.9% | 37.5% | 13.9% | 34.7% | 27.3% | 51.2% |
|  |  | NLPLE | 3.4% | 13.0% | 3.0% | 21.5% | 3.3% | 19.0% | 3.0% | 14.0% | 3.5% | 16.9% | 3.0% | 11.4% |
|  |  | LPLE | 56.0% | 78.3% | 54.9% | 91.2% | 64.2% | 94.9% | 55.6% | 87.5% | 59.1% | 88.4% | 48.0% | 64.5% |
| F | 70 | PFLE | 48.6% | 59.6% | 38.5% | 50.9% | 41.2% | 51.2% | 40.7% | 51.8% | 36.8% | 46.3% | 39.7% | 49.5% |
|  |  | NLPLE | 6.3% | 9.2% | 9.9% | 14.7% | 8.5% | 11.9% | 8.1% | 11.5% | 8.7% | 12.6% | 8.9% | 12.9% |
|  |  | LPLE | 34.8% | 41.8% | 38.9% | 47.3% | 39.9% | 48.0% | 39.9% | 47.5% | 43.6% | 52.3% | 40.6% | 48.3% |
|  | 80 | PFLE | 36.7% | 49.3% | 32.2% | 43.0% | 33.8% | 44.8% | 35.0% | 48.6% | 29.8% | 40.6% | 33.9% | 44.1% |
|  |  | NLPLE | 6.2% | 10.4% | 8.1% | 13.2% | 8.5% | 14.0% | 6.9% | 11.3% | 6.2% | 10.2% | 7.5% | 12.5% |
|  |  | LPLE | 44.7% | 53.9% | 47.9% | 57.3% | 46.8% | 55.7% | 45.6% | 54.4% | 52.2% | 62.4% | 47.5% | 56.3% |
|  | 90 | PFLE | 21.5% | 39.2% | 19.3% | 35.0% | 20.1% | 37.3% | 22.8% | 44.0% | 18.4% | 34.2% | 22.2% | 37.0% |
|  |  | NLPLE | 5.4% | 12.7% | 4.9% | 12.2% | 6.7% | 17.5% | 4.6% | 11.9% | 3.3% | 9.0% | 5.2% | 13.2% |
|  |  | LPLE | 54.2% | 73.0% | 56.6% | 74.5% | 53.2% | 71.9% | 52.4% | 68.3% | 59.7% | 79.6% | 54.5% | 73.1% |

Continued

Table e4: Continued

| Relative estimates | | | | | | | | | | | | |
| --- | --- | --- | --- | --- | --- | --- | --- | --- | --- | --- | --- | --- |
| Baseline = Limiting pain | | | | | | | | | | | | |
| Sex | Age | Est. | 2008 | | 2010 | | 2012 | | 2014 | | 2016 | |
|  |  |  | Lo | Hi | Lo | Hi | Lo | Hi | Lo | Hi | Lo | Hi |
| M | 70 | PFLE | 36.3% | 48.5% | 44.0% | 56.2% | 37.1% | 49.3% | 35.8% | 50.2% | 36.2% | 49.2% |
|  |  | NLPLE | 12.3% | 18.0% | 9.5% | 14.2% | 11.4% | 18.1% | 12.2% | 18.6% | 12.5% | 19.7% |
|  |  | LPLE | 38.2% | 48.4% | 34.3% | 41.9% | 37.8% | 47.3% | 37.2% | 47.0% | 35.4% | 45.7% |
|  | 80 | PFLE | 23.6% | 36.9% | 34.1% | 48.6% | 25.7% | 42.0% | 25.4% | 38.0% | 27.0% | 40.8% |
|  |  | NLPLE | 8.7% | 15.3% | 7.1% | 12.6% | 8.2% | 15.6% | 10.0% | 16.7% | 10.9% | 18.1% |
|  |  | LPLE | 52.6% | 66.7% | 44.4% | 55.9% | 50.5% | 61.9% | 50.5% | 61.1% | 47.3% | 59.2% |
|  | 90 | PFLE | 8.9% | 27.7% | 17.2% | 39.6% | 11.9% | 30.9% | 11.5% | 27.2% | 12.2% | 31.1% |
|  |  | NLPLE | 3.6% | 11.8% | 3.7% | 11.8% | 3.9% | 15.3% | 5.6% | 15.5% | 6.0% | 18.7% |
|  |  | LPLE | 64.6% | 90.4% | 53.5% | 80.3% | 60.9% | 87.1% | 62.6% | 86.1% | 59.5% | 82.3% |
| F | 70 | PFLE | 35.1% | 44.0% | 45.1% | 54.2% | 31.9% | 40.5% | 30.7% | 39.9% | 35.7% | 46.2% |
|  |  | NLPLE | 10.1% | 13.9% | 9.6% | 13.1% | 10.6% | 14.7% | 10.2% | 14.6% | 10.7% | 15.5% |
|  |  | LPLE | 45.0% | 52.0% | 36.3% | 42.5% | 46.4% | 55.3% | 47.6% | 56.5% | 42.4% | 51.2% |
|  | 80 | PFLE | 28.9% | 38.6% | 36.3% | 48.7% | 26.3% | 38.9% | 26.8% | 35.5% | 31.0% | 43.0% |
|  |  | NLPLE | 8.7% | 13.8% | 8.6% | 13.7% | 8.1% | 12.5% | 8.1% | 13.5% | 8.5% | 14.2% |
|  |  | LPLE | 51.4% | 60.9% | 43.0% | 51.8% | 51.4% | 64.2% | 54.3% | 63.1% | 47.7% | 57.1% |
|  | 90 | PFLE | 18.0% | 30.5% | 22.5% | 41.5% | 17.7% | 36.1% | 16.9% | 29.3% | 20.1% | 38.9% |
|  |  | NLPLE | 5.3% | 13.5% | 7.0% | 15.3% | 4.7% | 11.1% | 5.1% | 12.8% | 5.5% | 13.7% |
|  |  | LPLE | 58.3% | 79.2% | 50.8% | 69.5% | 57.6% | 80.7% | 59.6% | 78.7% | 55.1% | 71.4% |
